# Synthesis of crispine A analogues *via* an intramolecular Schmidt reaction

**DOI:** 10.1186/1860-5397-3-49

**Published:** 2007-12-19

**Authors:** Ajoy Kapat, Ponminor Senthil Kumar, Sundarababu Baskaran

**Affiliations:** 1 Department of Chemistry, Indian Institute of Technology Madras, Chennai-600 036, India

## Abstract

An intramolecular Schmidt reaction strategy for the synthesis of various derivatives of crispine A using azido-ketone as a key intermediate is described.

## Background

The indolizidine skeleton is one of the most important structural subunits present in numerous biologically active molecules. [1–4] The polyhydroxylated indolizidines are potent inhibitors of carbohydrate processing enzymes and hence they are considered to be lead drug molecules in the treatment of metabolic diseases such as diabetes, cancer and HIV infection. [5–7] The alkyl indolizidine alkaloids, also called gephyrotoxins, are well-known for their ability to function as *non-competitive blockers of neuromuscular transmission* [2] by interacting with nAChRs. In addition, the indolizidine skeleton is also present in anticancer molecules such as lepadiformine,[8] antofine,[9] and tylophorine [9] as well as a immunosuppressive agent, FR901483.[10] The wide range of biological activities associated with the indolizidine alkaloids has elicited considerable interest in them as target molecules among synthetic organic chemists. As a result, numerous synthetic approaches have been developed for the synthesis of indolizidine alkaloids. [5–7] One of the most efficient methods for the construction of the indolizidine framework is based on the intramolecular Schmidt reaction of azides with carbonyl compounds.[11–12] Pearson and Aube have exploited the synthetic potential of the intramolecular Schmidt reaction in the synthesis of several indolizidine alkaloids. [11–15]

**Scheme 1:**
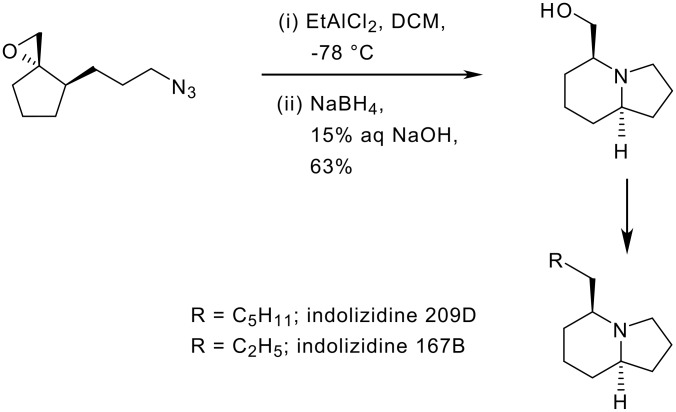
Epoxide initiated electrophilic cyclization of azide.

Recently, we reported a novel approach for the construction of the indolizidine skeleton using an epoxide initiated electrophilic cyclization of azide as a key step. This novel methodology has been efficiently applied in the stereo- and enantioselective synthesis of indolizidine 167B and 209D (Scheme 1). [16–18]

## Results and discussion

**Scheme 2:**
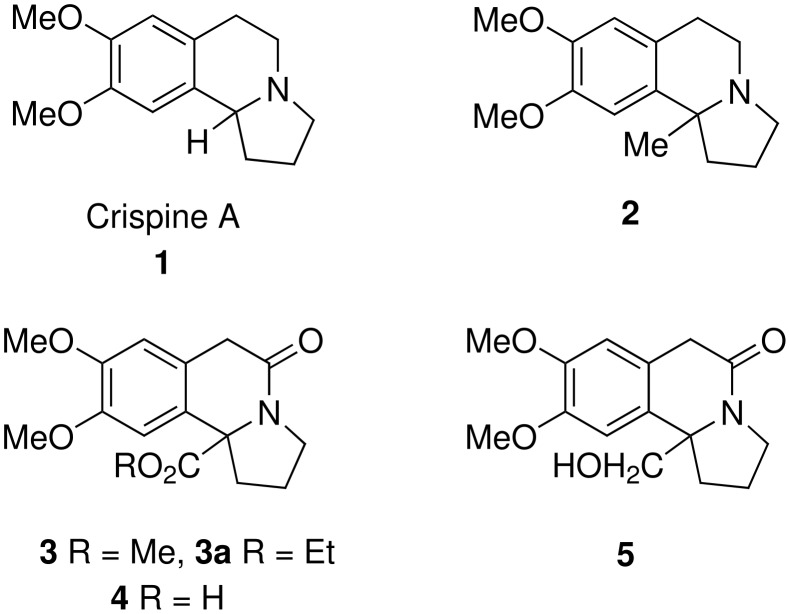
Crispine A and its analogues.

In 2002, a new indolizidine alkaloid known as crispine A was isolated from *Carduus crispus*, a popular invasive plant occurring in Asia and Europe, which was found to exhibit superior antitumor activity against SKOV3, KB and HeLa human cancer lines.[19] As a result of its potent antitumor activity, various synthetic methods have been developed for the synthesis of crispine A. [20–28] Interestingly, Schell and Smith reported the first synthesis of crispine A, even before its isolation, using the *N*-chloramine rearrangement reaction as a key step.[25] In order to understand the structure activity relationship (SAR) as well as to improve the efficacy of this novel anti-cancer agent, a flexible approach for the synthesis of various derivatives of crispine A is in great demand (Scheme 2).

**Scheme 3:**
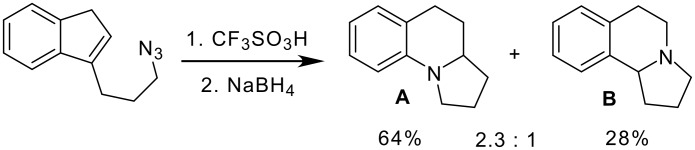
Intramolecular Schmidt reaction of olefin azide.

In 2000, Pearson reported the intramolecular Schmidt reaction based approach for the construction of benzo-fused indolizidine skeleton using azido-olefin as a key intermediate (Scheme 3). In this reaction, in addition to benzo[*e*]indolizidine **A**, a minor product **B** having the basic skeleton of crispine A was isolated in 28% yield. The intramolecular Schmidt reaction of azido-olefin in the presence of triflic acid proceeds with aryl migration rather than alkyl migration resulting in the formation of benzo[*e*]indolizidine [**A**] as a major product (Scheme 3).[29]

**Scheme 4:**
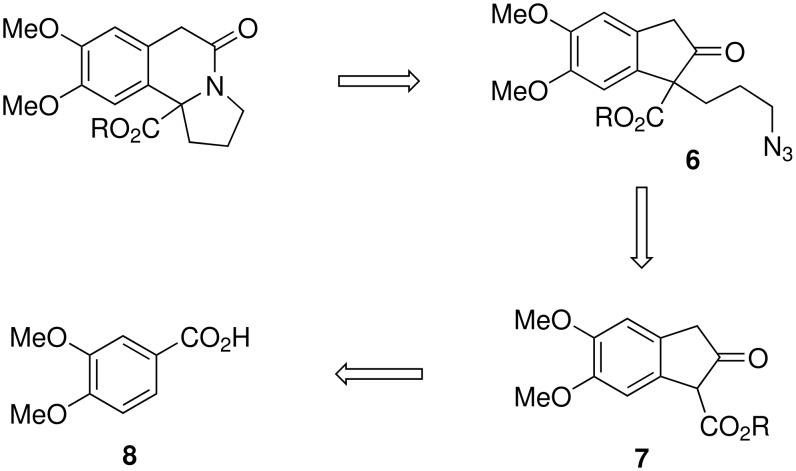
Retrosynthetic approach for crispine A analogues.

In this communication, we report the synthesis of crispine A analogues (**2–5**) using an intramolecular Schmidt reaction of azidoketone **6** as a key step. The azidoketone **6** can be readily prepared from the β-ketoester **7**, which in turn can be synthesized from the dimethoxybenzoic acid **8** as shown in Scheme 4.[30] 3,4-Dimethoxybenzoic acid (**8**) on treatment with paraformaldehyde in the presence of conc. H_2_SO_4_ followed by reduction with LAH gave the corresponding diol **9** as a white crystalline solid. Diol **9** on bromination followed by nucleophilic displacement with NaCN furnished the desired dicyano compound **10**.

**Scheme 5:**
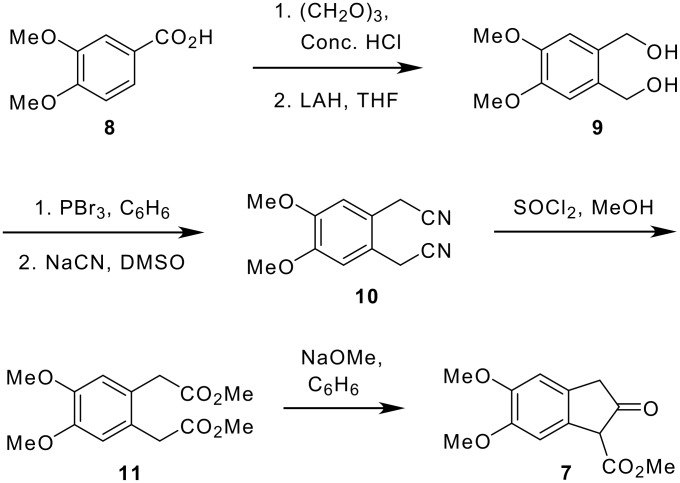
Synthesis of β-ketoester **7**.

Treatment of dicyanide **10** with thionyl chloride in methanol gave the corresponding diester **11** as a colorless liquid in good yield. Compound **11** was then readily converted to the corresponding β-ketoester **7** *via* Dieckmann cyclization and the resultant product was purified by recrystallization using H_2_O-EtOH solvent system (Scheme 5).

**Scheme 6:**
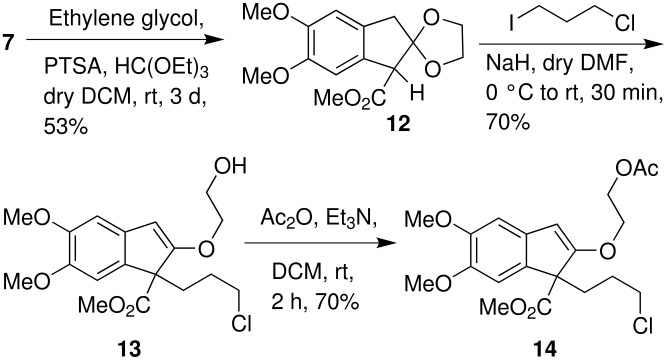
Alkylation of ketal-ester **12**.

Our attempts towards the alkylation of β-ketoester **7** with 1-chloro-3-iodopropane under different reaction conditions were ineffective and resulted in poor yield. In order to improve the yield of the alkylation reaction, compound **7** was protected as the corresponding ethylene ketal **12** (Scheme 6).

Surprisingly, alkylation of ketal-ester **12** using NaH in dry DMF proceeded smoothly even at room temperature, however it resulted in an unusual cleavage of ethylene ketal under basic conditions, leading to hydroxy vinylether **13** in 70% yield. The formation of hydroxy vinylether **13** is evident from the spectroscopic data. The presence of a sharp singlet at δ_H_ 5.66 (s,1H) in ^1^H NMR and signals corresponding to vinyl carbons (δ_c_ 104.28, 164.39) in ^13^C NMR, as well as an absorption at 3513 cm^-1^ in IR spectrum, clearly indicate the presence of a vinylether and a free hydroxyl group in compound **13**. Reaction of hydroxy vinylether **13** with acetic anhydride yielded readily the corresponding acetate derivative **14** which further supported the formation of hydroxy vinylether under basic conditions (Scheme 6).

**Scheme 7:**
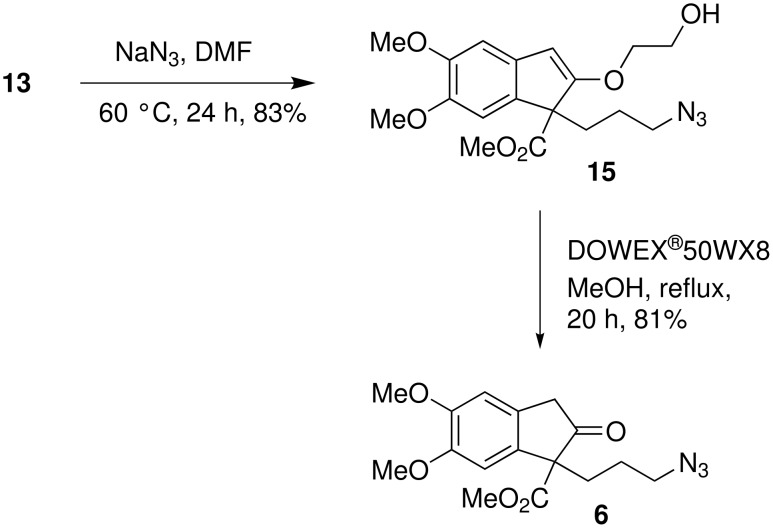
Synthesis of azido-ketone **6**.

Reaction of **13** with NaN_3_ gave the corresponding azido derivative **15** which on further treatment with DOWEX^®^50WX8H^+^ in methanol under reflux conditions afforded the corresponding azido-ketone **6** in 81% yield (Scheme 7).

**Scheme 8:**
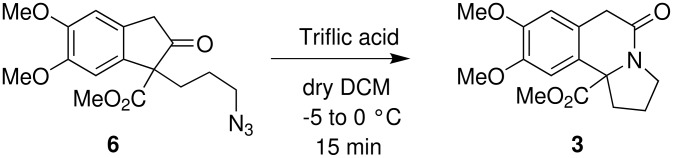
The intramolecular Schmidt cyclization of azido-ketone **6**.

Finally, the intramolecular Schmidt reaction of azido-ketone **6** was successfully achieved using TfOH at -5 to 0°C and the resultant cyclized product, indolizidine derivative **3**, was isolated in 54% yield (Scheme 8). Similarly, the indolizidine derivative **3a** was prepared from the dicyanide **10**.

**Figure 1:**
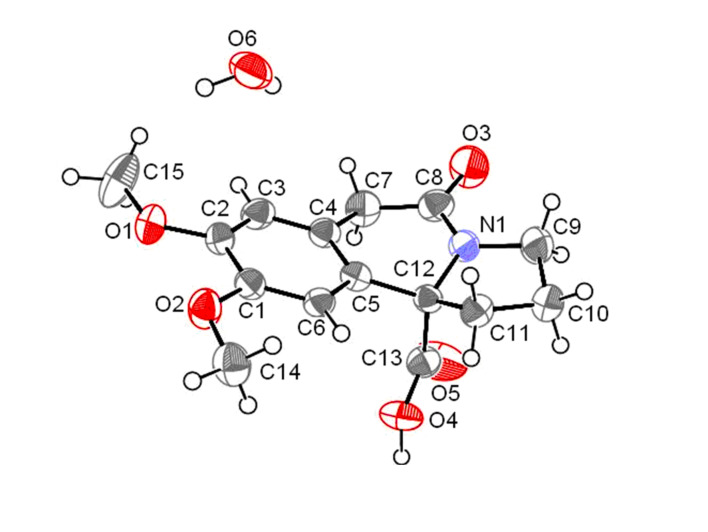
ORTEP diagram of the acid derivative **4**.

**Scheme 9:**
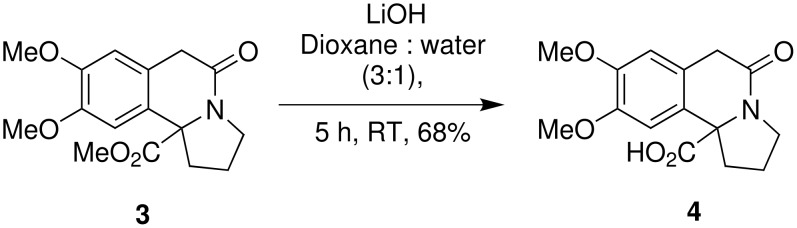
Synthesis of acid analogue of crispine A.

The structure of indolizidine derivative **3** was established by 1D and 2D NMR analyses which was unambiguously further confirmed by single crystal X-ray analysis (Figure 1), on the corresponding acid derivative **4** (Scheme 9).

**Scheme 10:**
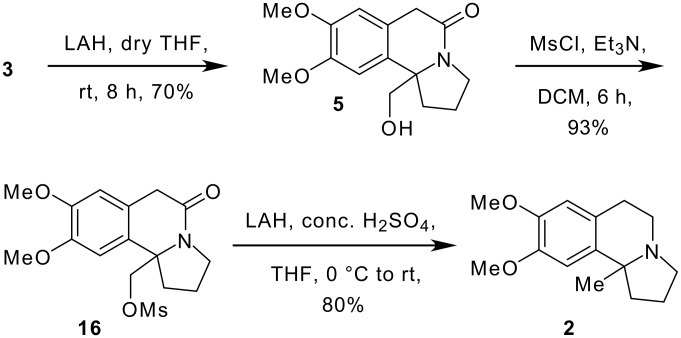
Synthesis of methyl analogue of crispine A.

After achieving the construction of the indolizidine skeleton using the intramolecular Schmidt reaction, our next objective was to prepare various derivatives of the anti-cancer agent, crispine A, starting from the key intermediate **3**. Consequently, the ester functional group of the indolizidine derivative **3** was reduced with LAH in dry THF at 0°C to give the corresponding hydroxymethyl derivative **5**. Mesylation of **5** with mesyl chloride and triethylamine yielded the corresponding lactam **16** which on further exposure to LAH in the presence of conc. H_2_SO_4_ [20] gave the methyl analogue of crispine A (**2**) in 80% yield (Scheme 10). Spectral data of compound **2** were found to be in complete agreement with the reported values.[26] (See Supporting Information File 1 for full experimental data)

## Conclusion

In conclusion, we have successfully achieved the synthesis of various derivatives of crispine A (**2–5**), starting from the azido ketone **6**, using the intramolecular Schmidt reaction as a key step. The structure of the cyclized indolizidine derivative **3** was unambiguously confirmed by single crystal X-ray analysis. Interestingly, an unusual cleavage of ethylene ketal to vinylether was observed during the alkylation of ketal-ester **12**. Since the compounds **5** and **16** are highly functionalized intermediates, they can be further exploited in the synthesis of a library of anti-cancer analogues. The structure activity relationships (SAR) and anti-cancer activities of our synthetic derivatives will be reported in due course of time.

## Supporting Information

File 1 Experimental section. Experimental data, which includes experimental procedures and spectral data.
