# Supplementary material for: Synthesis of crispine A analogues via an intramolecular Schmidt reaction
Source: Beilstein J Org Chem. 2007 Dec 19;3:49. doi: 10.1186/1860-5397-3-49 (PMC2213660; doi:10.1186/1860-5397-3-49)
Supplement: File 1 — Experimental section. Experimental data, which includes experimental procedures and spectral data. [file Beilstein_J_Org_Chem-03-49-s001.pdf]

Supporting Information

Synthesis of Crispine-A analogues *via* an intramolecular Schmidt reaction

*Ajoy Kapat, Ponminor SenthilKumar and Sundarababu Baskaran,**

Department of Chemistry, Indian Institute of Technology Madras, Chennai-600 036, India

Corresponding Author E-*mail*: *sbhaskar@iitm.ac.in*

Experimental Section………………1 – 15
Single crystal X-ray analysis ………………10
NMR Spectra for selected compounds……………....16 - 42

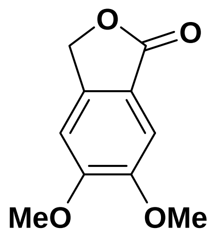

5,6-dimethoxyisobenzofuran-1(3H)-one ^{1}H NMR [400 MHz, $CDCl_3$] δ 7.27 (s, 1H), 6.87 (s, 1H), 5.19 (s, 2H), 3.96 (s, 3H), 3.93 (s, 3H), ^{13}C NMR [100 MHz, $CDCl_3$] δ 171.4, 154.8 150.4, 141.0, 117.6, 103.4, 69.1, 56.3, 56.2; MS (ESI) $C_{10}H_{11}O_4$ $(M+H)^+$ 195.

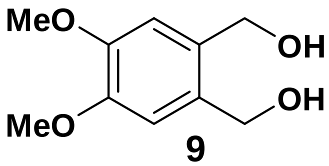

9

1,2-Bis(hydroxymethyl)-4,5-dimethoxybenzene (9): To a stirred solution of LAH (430 mg, 11.34 mmol) in dry THF (20 ml) at 0 °C was slowly added a solution of 5,6-dimethoxyisobenzofuran-1(3H)-one (2 g, 10.306 mmol) and stirred at 0 °C for 15 mins. The reaction mixture was then heated to reflux for 4h, cooled to 0 °C, quenched with moist Na_2SO_4 and filtered. The filtrated was then concentrated under reduced pressure to give the corresponding diol (**9**) (1.92 g, 94% yield). The crude product was taken for further reaction without purification. (IR (Neat): 3460, 3337, 2922, 1607, 1511, 1460, 1103, 754 cm^{-1}; ^{1}H NMR [400 MHz, $CDCl_3$] δ 6.89 (s, 2H), 4.66 (s, 4H), 3.89 (s,

6H), 3.08 (bs, 1H), 1.76 (bs, 1H), ^{13}C NMR [100 MHz, $CDCl_3$] δ 148.5, 132.0, 113.2, 63.8, 56.0; MS (ESI) $C_{10}H_{14}O_4$ $(M+Na)^+$ 221.

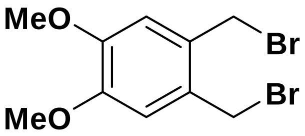

1,2-Bis(bromomethyl)-4,5-dimethoxybenzene: To a suspension of diol (**9**) (2 g, 10.10 mmol) in benzene (20 ml) was slowly added PBr_3 (2.06 ml, 22 mmol) and stirred at 50 °C for 1h then cooled to rt and stirred for 24h. The reaction mixture was then basified with saturated Na_2CO_3 solution (PH = 9). Organic layer was washed with water and dried over anhydrous Na_2SO_4, concentrated under reduced pressure and purified by recrystallization to afford the corresponding **1,2-bis(bromomethyl)-4,5-dimethoxybenzene** (3.01 g, 92% yield). IR (neat): 2955, 1605, 1522, 1460, 1358, 1277, 1204, 1128, 1000, 871, 603 cm^{-1}; ^1H NMR [400 MHz, $CDCl_3$] δ 6.84 (s, 2H), 4.63 (s, 4H), 3.89 (s, 6H); ^{13}C NMR [100 MHz, $CDCl_3$] δ 149.5, 129.0, 113.7, 56.0, 30.5.

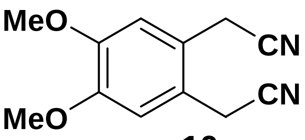

10

2,2'-(4,5-dimethoxy-1,2-phenylene)diacetonitrile (10): To a stirred solution of NaCN (3.16 g, 64.51 mmol) in dry DMSO (50 ml) was slowly added a solution of 1,2-bis(bromomethyl)-4,5-dimethoxybenzene (9.5 g, 29.321

mmol) at 90 °C. The reaction mixture was stirred at 90 °C for additional 3h. Then quenched with water and extracted with chloroform. Organic layer was dried over anhydrous Na_2SO_4, concentrated under reduced pressure and purified by column chromatography over silica gel using gradient elution with 10 – 20 % EtOAc in hexane to yield the corresponding dicyano compound (**10**) (5.71 g, 90% yield). IR (neat): 2959, 2941, 2837, 2250, 1609, 1525, 1417, 1272, 1180, 1089, 873, 756 cm^{-1}; 1H NMR [400 MHz, $CDCl_3$] δ 6.90 (s, 2H), 3.90 (s, 6H), 3.70 (s, 4H); ^{13}C NMR [100 MHz, $CDCl_3$] δ 149.3, 120.2, 116.9, 112.8, 56.1, 21.0; MS (ESI) $C_{12}H_{12}N_2O_2$ $(M+Na)^+$ 239.

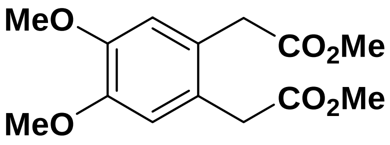

11

Dimethyl 2,2'-(4,5-dimethoxy-1,2-phenylene)diacetate (11a): To a solution of dicyano compound (**10**) (2.12 g, 8.62 mmol) in methanol (15 ml), thionyl chloride was slowly added (4.13 ml, 56.96 mmol) and heated to reflux for 2 days. Reaction mixture was diluted with EtOAc and washed with water. Organic layer was dried over anhydrous Na_2SO_4, concentrated and purified by column chromatography over silica gel using gradient elution with 10 – 20 % EtOAc in hexane to yield the corresponding diester (**11**) (2.86 g, 94% yield). IR (neat): 2952, 2851, 1729, 1520, 1273, 1192, 1009, 754 cm^{-1}; ^1H NMR

[400 MHz, $CDCl_3$] δ 6.76 (s, 2H), 3.85 (s, 6H), 3.67 (s, 6H), 3.62 (s, 4H); ^{13}C NMR [100 MHz, $CDCl_3$] δ 171.7, 148.3, 125.2, 114.1, 55.9, 51.8, 38.2, 29.60; MS (ESI) $C_{14}H_{18}O_6$ $(M+Na)^+$ 305.

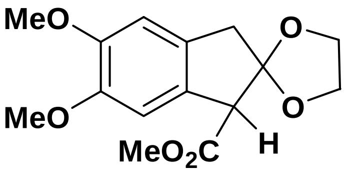

12

Methyl 5',6'-dimethoxy-1',3'-dihydrospiro[[1,3]dioxolane-2,2'-indene]-1'-carboxylate (12): To a stirred solution of β-ketoester (**7**) (100 mg, 0.378 mmol), triethyl orthoformate (251 µl, 1.51 mmol) and ethylene glycol (212 µl, 3.77 mmol) in dry DCM (10 ml) added catalytic amount of PTSA (7 mg, 0.03 mmol) and the reaction mixture was stirred for about 3 days. Reaction mixture was then diluted with DCM, washed with saturated $NaHCO_3$ followed by brine and extracted with DCM. Organic layer was dried over anhydrous Na_2SO_4, conc. and purified by column chromatography over silica gel using gradient elution with 10 – 20 % EtOAc in hexane to yield the corresponding ethylene ketal (**12**) (53%).IR (neat): 2957, 2836, 1728, 1508, 1466, 1257, 1180, 1031, 857, 572 cm^{-1}; ^1H NMR [400 MHz, $CDCl_3$] δ 6.77 (s, 1H), 6.74 (s, 1H), 4.01-4.11 (m, 4H), 3.86 (s, 3H), 3.84 (s, 3H), 3.73 (s, 3H), 3.73 (s, 3H), 3.43 (d, J = 16 Hz, 1H), 3.06 (d, J = 16 Hz, 1H); ^{13}C NMR [100 MHz,

CDCl3] δ 171.0, 149.4, 148.5, 132.3, 129.4, 117.9, 108.3, 107.7, 65.4, 64.5, 59.3, 56.0, 55.9, 52.0, 42.9; MS (ESI) $C_{15}H_{18}O_6$ $(M+Na)^+$ 317.

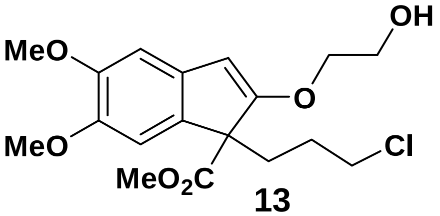

13

Methyl 1-(3-chloropropyl)-2-(2-hydroxyethoxy)-5,6-dimethoxy-1H-indene-1-carboxylate (13): To a suspension of NaH (14 mg, 0.35 mmol) in dry DMF (3 ml) at 0 °C was slowly added a solution of ethylene ketal (**12**) (100 mg, 0.325 mmol) in dry DMF (3 ml) and stirred at 0 °C for 5 min. Then into this added 1-chloro-3-iodopropane (52 µl, 0.487 mmol) and stirred at 0 °C for 30 min. The reaction mixture was then quenched, washed with water and extracted with EtOAc. Organic layer was dried over anhydrous Na_2SO_4, concentrated under reduced pressure and purified by column chromatography over silica gel using gradient elution with 10 – 20 % EtOAc in hexane to yield the alkylated product (**13**) (87.3 mg, 70% yield). IR (neat): 3513, 2951, 2835, 1727, 1607, 1491, 1454, 1312, 1213, 1075, 1032, 761 cm^{-1}; ^1H NMR [400 MHz, $CDCl_3$] δ 6.86 (s, 1H), 6.72 (s, 1H), 5.66 (s, 1H), 4.15-4.10 (m, 4H), 3.87 (s, 3H), 3.86 (s, 3H), 3.67 (s, 3H), 3.42-3.37 (m, 2H), 2.32 (t, *J* = 8 Hz,

1H), 1.46-1.25 (m, 2H); ^{13}C NMR [100 MHz, $CDCl_3$] δ 172.6, 164.3, 149,4, 146.1, 136.2, 129.9, 107.2, 104.0, 101.2, 71.5, 60.7, 60.4, 56.6.

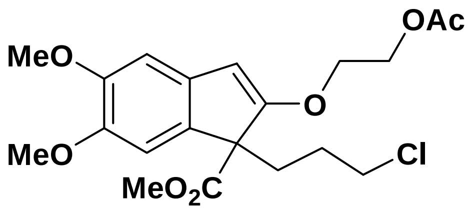

14

Methyl 1-(3-chloropropyl)-2-(2-hydroxyethoxy)-5,6-dimethoxy-1H-indene-1-carboxylate (14): To a stirred solution of alkylated compound (13) (9 mg, 0.024 mmol) in dry DCM (1 ml), acetic anhydride (3.47 µl, 0.0364 mmol) and triethyl amine (3.23 µl, 0.0243 mmol) was added followed by catalytic amount DMAP and stirred at rt for 2h. The reaction mixture was then diluted with DCM and washed with water. Organic layer was dried over anhydrous Na_2SO_4, conc. under reduced pressuren and purified by column chromatography over silica gel using gradient elution with 10-15 % EtOAc in hexane to yield the acetylated compound (**14**) (7 mg, 70% yield). IR (neat): 2952, 1731, 1608, 1491, 1455, 1316, 1232, 1058, 761 cm^{-1}; ^{1}H NMR [400 MHz, $CDCl_3$] δ 6.83 (s, 1H), 6.73 (s, 1H), 5.64 (s, 1H), 4.41-4.39 (m, 2H) 4.20-4.13 (m, 2H), 3.87 (s, 3H), 3.86 (s, 3H), 3.63 (s, 3H), 3.41-3.36 (m, 2H), 2.32-2.27 (m, 2H), 2.09 (s, 3H), 1.29-1.25 (m, 2H); ^{13}C NMR [100 MHz,

$CDCl_3$] δ 172.4, 170.8, 164.8, 149.5, 146.2, 136.1, 130.4, 107.1, 104.2, 100.9, 68.09, 62.1, 61.1, 56.6, 56.1, 52.6, 44.9, 30.5, 26.6, 20.7; MS (ESI) $C_{20}H_{25}ClO_7$ $(M+H)^+$ 413.

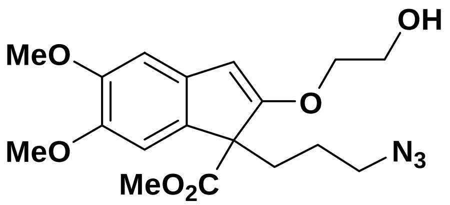

15

Methyl 1-(3-azidopropyl)-2-(2-hydroxyethoxy)-5,6-dimethoxy-1H-indene-1-carboxylate (15): To a solution of alkylated compound (**13**) (70 mg, 0.0189 mmol) in DMF (2 ml) was added NaN_3 (13.5 mg, 0.020 mmol) and stirred at 60 °C for 24h. Reaction mixture was then diluted with EtOAc, washed with water. Organic layer was dried, conc. and purified by column chromatography over silica gel using gradient elution with 10 – 20 % EtOAc in hexane to yield the corresponding azido compound (**15**) (59 mg, 83% yield). IR (neat): 3498, 2935, 2094, 1727, 1607, 1491, 1455, 1309, 1214, 1966, 762 cm^{-1}; ^1H NMR [400 MHz, $CDCl_3$] δ 6.85 (s, 1H), 6.73 (s, 1H), 5.67 (s, 1H), 4.11-3.91 (m, 4H), 3.87 (s, 3H), 3.86 (s, 3H), 3.67 (s, 3H), 3.18-3.14 (m, 2H), 2.27-2.23 (m, 2H), 1.29-1.25 (m, 2H); ^{13}C NMR [100 MHz, $CDCl_3$] δ 172.5, 164.5, 149.7, 146.3, 136.3, 130.1, 107.6, 104.4, 100.2, 71.6, 62.3, 60.6, 56.7, 56.1, 52.6, 51.2, 30.3, 22.7.; MS (ESI) $C_{18}H_{23}N_3O_6$ $(M+Na)^+$ 400.

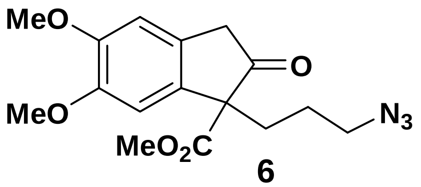

6

Methyl 1-(3-azidopropyl)-5,6-dimethoxy-2-oxo-2,3-dihydro-1H-indene-1-carboxylate (6): To a stirred solution of azide (**15**) (488 mg, 1.294 mmol) in methanol (10 ml) was added Dowex activated using 1N HCl and heated to reflux for 20h. The reaction mixture was then filtered, concentrated under reduced pressure and the residue was purified by column chromatography over silica gel using gradient elution with 10 – 20 % EtOAc in hexane to yield the corresponding keto azide (**6**) (349 mg, 81% yield). IR (neat): 2913, 2095, 1613, 1494, 1454, 1286, 1164, 1032, 763 cm^{-1}; ^{1}H NMR [400 MHz, $CDCl_3$] δ 6.87 (s, 1H), 6.76 (s, 1H), 3.90 (s, 3H), 3.89 (s, 3H), 3.76 (d, J = 22.4 Hz, 1H), 3.65 (s, 3H), 3.44 (d, J = 22.4 Hz), 3.24-3.16 (m, 2H), 2.28-2.19 (m, 2H), 1.31-1.26 (m, 2H); ^{13}C NMR [100 MHz, $CDCl_3$] δ 212.2, 170.8, 149,9, 145.5, 131.6, 129.1, 107.7, 106.4, 64.6, 56.1, 56.0, 52.8, 51.1, 43.4, 31.0, 23.7.

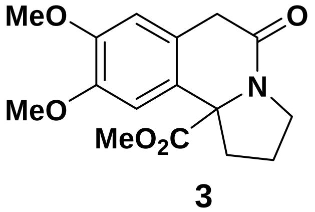

3

Methyl 8,9-dimethoxy-5-oxo-1,2,3,5,6,10b-hexahydropyrrolo[2,1-a]isoquinoline-10b-carboxylate (3): In a clean flame dried RB flask, ketoazide (**15**) (364 mg, 1.04 mmol) was taken and dried azeotropically with dry benzene. Into this added DCM (5 ml) and cooled to -5 °C. Triflic acid (139 μl, 1.57 mmol) was slowly added and stirred at -5 °C for 15min. Reaction mixture was then diluted with DCM and washed with water. Organic layer was dried over anhydrous Na_2SO_4 concentrated under reduced pressure and purified by column chromatography over silica gel using gradient elution with 30 – 40 % EtOAc in hexane to yield the corresponding cyclized product (**3**) (180.69 mg, 54% yield). IR (neat): 2953, 1731, 1650, 1518, 1433, 1411, 1254, 1217, 1133, 805, 628 cm^{-1}; ^1H NMR [400 MHz, $CDCl_3$] δ 6.77 (s, 1H), 6.55 (s, 1H), 3.83 (s, 3H), 3.80 (s, 3H), 3.70 (d, *J* = 19.2 Hz, 1H), 3.59 (s, 3H), 3.58-3.56 (m, 1H), 3.41 (d, *J* = 18.8 Hz, 1H), 3.05-3.02 (m, 1H), 2.05-1.97 (m, 2H), 1.88-1.81 (m, 2H); ^{13}C NMR [100 MHz, $CDCl_3$] δ 172.1, 168.1, 149.4, 148.1, 126.0, 125.1, 110.0, 108.6, 71.6, 56.2, 56.0, 53.1, 44.7, 37.6, 36.2, 21.5; HRMS (ESI) calcd for $C_{16}H_{19}NO_5$ $(M+H)^+$: 306.134; found: 306.1350.

Single Crystal X-Ray Analysis of Hydroxy analogue of Crispine A (4):

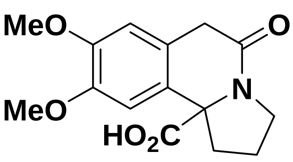

4

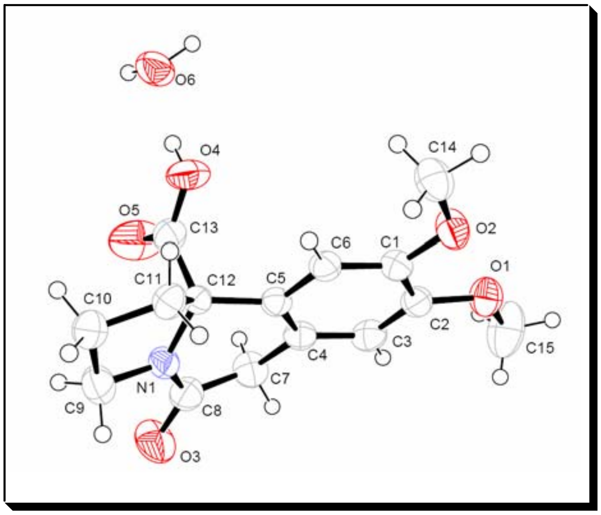

To a solution of ester (**3**) (40 mg, 0.125 mmol) in dioxane **:** water (3:1) (3 ml), KOH was added and stirred at rt for 5h. Then diluted with water and neutralized (PH = 6) with 1N HCl and extracted with EtOAc. Organic layers were dried over anhydrous Na_2SO_4, concentrated under reduced pressure and purifired by recrystallisation from EtOAc:Hexane (1:5) (5 mg/mL) afforded suitable size and quality crystals for x-ray diffraction in the form of pale yellow needles. CCDC Number: CCDC 663178

Table 1. Crystal data and structure refinement for ajoy.

Identification code	ajoy
Empirical formula	C15 H19 N O6
Formula weight	309.31
Temperature	298(2) K
Wavelength	0.71073 A

Crystal system, space group	Orthorhombic, P2(1)2(1)2(1)
Unit cell dimensions	a = 10.3243(9) A alpha = 90 deg.
	b = 10.6834(10) A beta = 90 deg.
	c = 13.6598(12) A gamma = 90 deg.
Volume	1506.7(2) A^3
Z, Calculated density	4, 1.364 Mg/m^3
Absorption coefficient	0.106 mm^-1
F(000)	656
Crystal size	0.25 x 0.22 x 0.22 mm
Theta range for data collection	2.42 to 28.46 deg.
Limiting indices	-13<=h<=9, -12<=k<=14, -17<=l<=14
Reflections collected / unique	19002 / 3594 [R(int) = 0.0738]
Completeness to theta = 25.00	100.0 %
Absorption correction	None
Max. and min. transmission	0.9771 and 0.9740
Refinement method	Full-matrix least-squares on F^2
Data / restraints / parameters	3594 / 3 / 214
Goodness-of-fit on F^2	0.740
Final R indices [I>2sigma(I)]	R1 = 0.0481, wR2 = 0.1125
R indices (all data)	R1 = 0.1352, wR2 = 0.1597

Absolute structure parameter -0.3(19)

Extinction coefficient 0.009(3)

Largest diff. peak and hole 0.151 and -0.167 e.A^-3

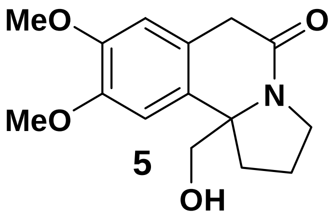

10b-(hydroxymethyl)-8,9-dimethoxy-1,2,3,10b-tetrahydropyrrolo[2,1-a]isoquinolin-5(6H)-one (5): To a suspension of LAH (7.5 mg, 0.19 mmol) in dry THF (2 ml) at 0 °C, a solution of ester (**3**) (55 mg, 0.18 mmol) in dry THF (2 ml) was slowly added and stirred at 0 °C for 8h. Reaction mixture was then quenched with moist Na_2SO_4. The solid was filtered, washed with EtOAc. Filtrate was concentrated under reduced pressure and purified by column chromatography over silica gel using gradient elution with 30–50 % EtOAc in hexane to yield the corresponding alcohol (5) (4.8 mg, 70 % yield). IR (neat): 3385, 2940, 1618, 1514, 1452, 1298, 1214, 1065, 765 cm^{-1}; ^1H NMR [400 MHz, $CDCl_3$] δ 6.67 (s, 1H), 6.64 (s, 1H), 3.88 (s, 3H), 3.86 (s, 3H), 3.78 (d, J = 19.2 Hz, 1H), 3.86-3.59 (m, 4H), 3.43 (d, J = 19.2 Hz, 1H), 2.60 (bs, 1H), 2.55-2.54 (m, 1H), 2.11-2.05 (m, 4H); ^{13}C NMR [100 MHz, $CDCl_3$] δ 168.5, 148.7, 147.9, 129.4, 124.9, 110.2, 108.4, 69.3, 67.9, 60.4,

56.2, 56.0, 44.9, 37.5, 33.8, 29.6, 21.2, 21.0, 14.1; MS (ESI) $C_{15}H_{19}NO_4$ $(M+H)^+$ 278.

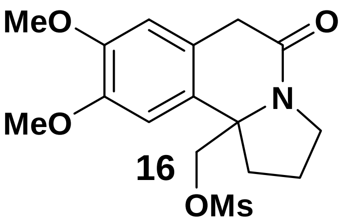

(8,9-dimethoxy-5-oxo-1,2,3,5,6,10b-hexahydropyrrolo[2,1-a]isoquinolin-10b-yl)methyl methanesulfonate (16): To a solution of alcohol (**5**) (5 mg, 0.0180 mmol) in dry DCM at 0 °C was added triethyl amine (5.5 µl, 0.03969 mmol) and stirred for 10 mins. Into this slowly added methane sulfonylchloride (1.54 µl, 0.0198 mmol) and stirred at 0 °C for 20 min. The temperature was then slowly raised to rt and stirred for 6h. Reaction mixture was then diluted with DCM and washed with water. Organic layer was dried over anhydrous Na_2SO_4, conc. and purified by column chromatography over silica gel using gradient elution with 30–50 % EtOAc in hexane to yield the corresponding mesylate (**16**) (6 mg, 93 % yield). IR (neat): 2940, 1618, 1514, 1452, 1366, 1298, 1214, 1065, 765 cm^{-1}; ^{1}H NMR [400 MHz, $CDCl_3$] δ 6.68 (s, 1H), 6.65 (s, 1H), 4.15 (dd, *J* = 23.2, 10 Hz, 2H), 3.89 (s, 3H), 3.88 (s, 3H), 3.77 (d, *J* = 19.6 Hz, 1H), 3.73-3.69 (m, 1H), 3.49 (d, *J* = 19.6 Hz, 1H), 2.82 (s, 3H), 2.64-2.62 (m, 1H), 2.22-2.12 (m, 2H), 1.43-1.37 (m, 2H); ^{13}C NMR

[100 MHz, $CDCl_3$] δ 168.1, 149.4, 148.3, 128.3, 127.8, 124.9, 110.4, 108.9, 72.4, 67.0, 56.4, 56.1, 46.2, 45.2, 37.5, 34.6, 31.5, 21.1; HRMS (ESI) calcd for $C_{16}H_{21}NO_6S$ $(M+Na)^+$: 378.0987; found: 378.0988.

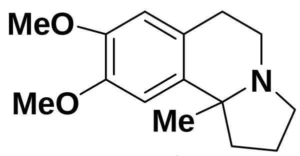

2

Pyrroridino[a]-1-methyl-1,2,3,4-terahydroisoquinoline (2):[26] To a stirred solution of LAH (1 mg, 0.0264 mmol) in dry THF (25 µl)at 0 °C under argon atmosphere was slowly added a solution of Conc.H_2SO_4 (0.5 µl) in 50 µl of dry THF. After stirring at 0 °C for 15 min, compound (**16**) (2 mg, 0.0072 mmol) in dry THF (20 µl) was added in one portion and the reaction was stirred at room temperature for 24 h. The reaction mixture was then cooled to 0 °C and 2 N NaOH (6 µl) was then carefully added and the reaction mixture stirred for 15 min. The solid was collected and washed with ether. Organic layer was dried over anhydrous Na_2SO_4, conc. under reduced pressure and purified by column chromatography over deactivated silica gel using gradient elution with 30–50% methanol in DCM to yield the corresponding methyl analogue ofCrispine A (**2**) in 80% yield. IR (neat) 2962, 2931, 2862, 1609, 1510, 1464, 1357, 1254, 1211, 1167, 1078, 996, 858, 770 cm-1; ^1H NMR [400 MHz, $CDCl_3$] δ 6.65 (s, 1H), 6.53 (s, 1H), 3.86 (s, 3H), 3.84 (s, 3H), 3.22

(ddd, J = 5.1, 11.4, 13.2 Hz, 1H), 3.12-2.80 (m, 4H), 2.45 (ddd, J = 15.9, 4.2, 2.6 Hz, 1H), 2.18-2.00 (m, 2H), 1.92-1.58 (m, 2H), 1.36 (s, 3H); HRMS (ESI) calcd for $C_{15}H_{21}NO_2$ $(M+Na)^+$: 248.1651; found: 248.1657.

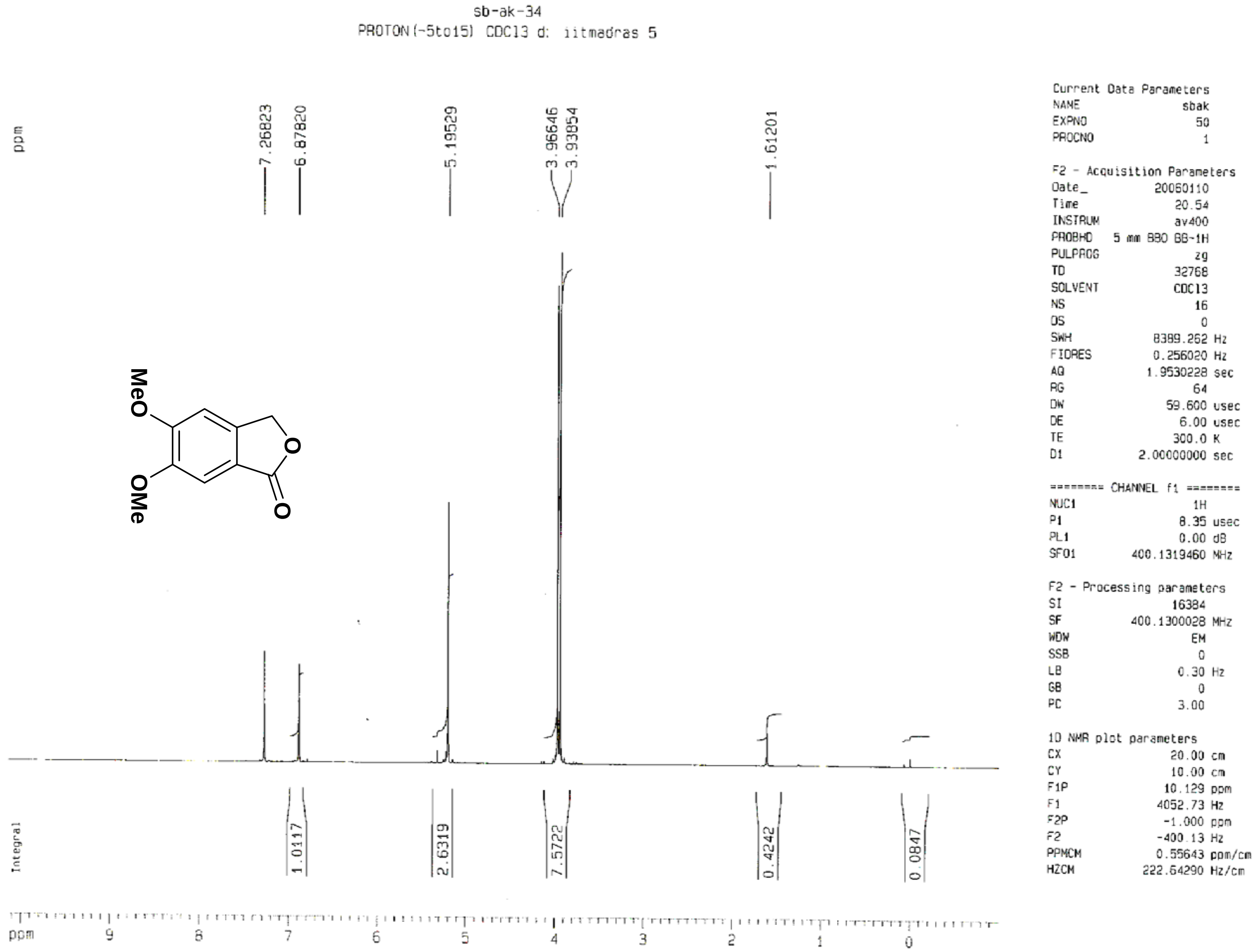
sb-ak-34
PROTON(-5to15) CDCl3 d: iitmadras 5
7.26823
6.87820
5.19529
3.96646
3.93854
1.61201
ppm
MeO
OMe
O
O
Integral
1.0117
2.6319
7.5722
0.4242
0.0847
ppm 9 8 7 6 5 4 3 2 1 0
Current Data Parameters
NAME sbak
EXPNO 50
PROCNO 1
F2 - Acquisition Parameters
Date_ 20060110
Time 20.54
INSTRUM av400
PROBHD 5 mm BBO BB-1H
PULPROG zg
TD 32768
SOLVENT CDCl3
NS 16
DS 0
SWH 8389.262 Hz
FIDRES 0.256020 Hz
AQ 1.9530228 sec
RG 64
DW 59.600 usec
DE 6.00 usec
TE 300.0 K
D1 2.00000000 sec
======== CHANNEL f1 ========
NUC1 1H
P1 8.35 usec
PL1 0.00 dB
SFO1 400.1319460 MHz
F2 - Processing parameters
SI 16384
SF 400.1300028 MHz
WDW EM
SSB 0
LB 0.30 Hz
GB 0
PC 3.00
1D NMR plot parameters
CX 20.00 cm
CY 10.00 cm
F1P 10.129 ppm
F1 4052.73 Hz
F2P -1.000 ppm
F2 -400.13 Hz
PPMCM 0.55643 ppm/cm
HZCM 222.64290 Hz/cm

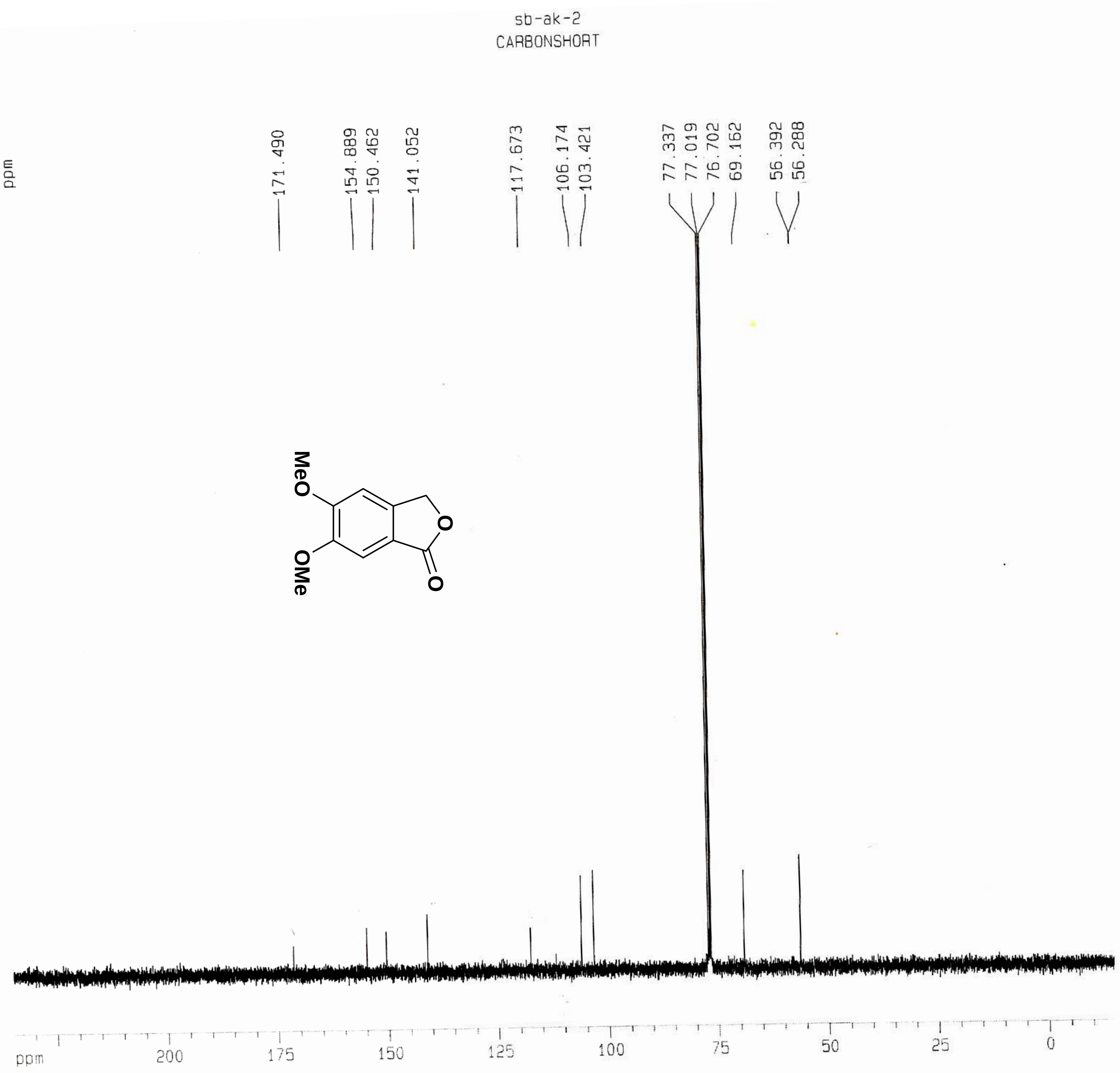

Current Data Parameters

NAME	sb
EXPNO	452
PROCNO	1

F2 - Acquisition Parameters

Date_	20050527
Time	18.58
INSTRUM	av400
PROBHD	5 mm BBO BB-1H
PULPROG	zgdc30
TD	65536
SOLVENT	CDCl3
NS	303
DS	2
SWH	25125.629 Hz
FIDRES	0.383387 Hz
AQ	1.3042164 sec
RG	90.5
DW	19.900 usec
DE	6.00 usec
TE	300.0 K
D1	2.00000000 sec
d11	0.03000000 sec

======== CHANNEL f1 ========

NUC1	13C
P1	5.50 usec
PL1	0.00 dB
SFO1	100.6238364 MHz

======== CHANNEL f2 ========

CPDPRG2	waltz16
NUC2	1H
PCPD2	80.00 usec
PL2	120.00 dB
PL12	18.00 dB
SFO2	400.1320007 MHz

F2 - Processing parameters

SI	131072
SF	100.6127690 MHz
WDW	EM
SSB	0
LB	1.00 Hz
GB	0
PC	1.40

1D NMR plot parameters

CX	20.00 cm
CY	14.00 cm
F1P	234.849 ppm
F1	23628.84 Hz
F2P	-14.877 ppm
F2	-1496.79 Hz
PPMCM	12.48630 ppm/cm
HZCM	1256.28137 Hz/cm

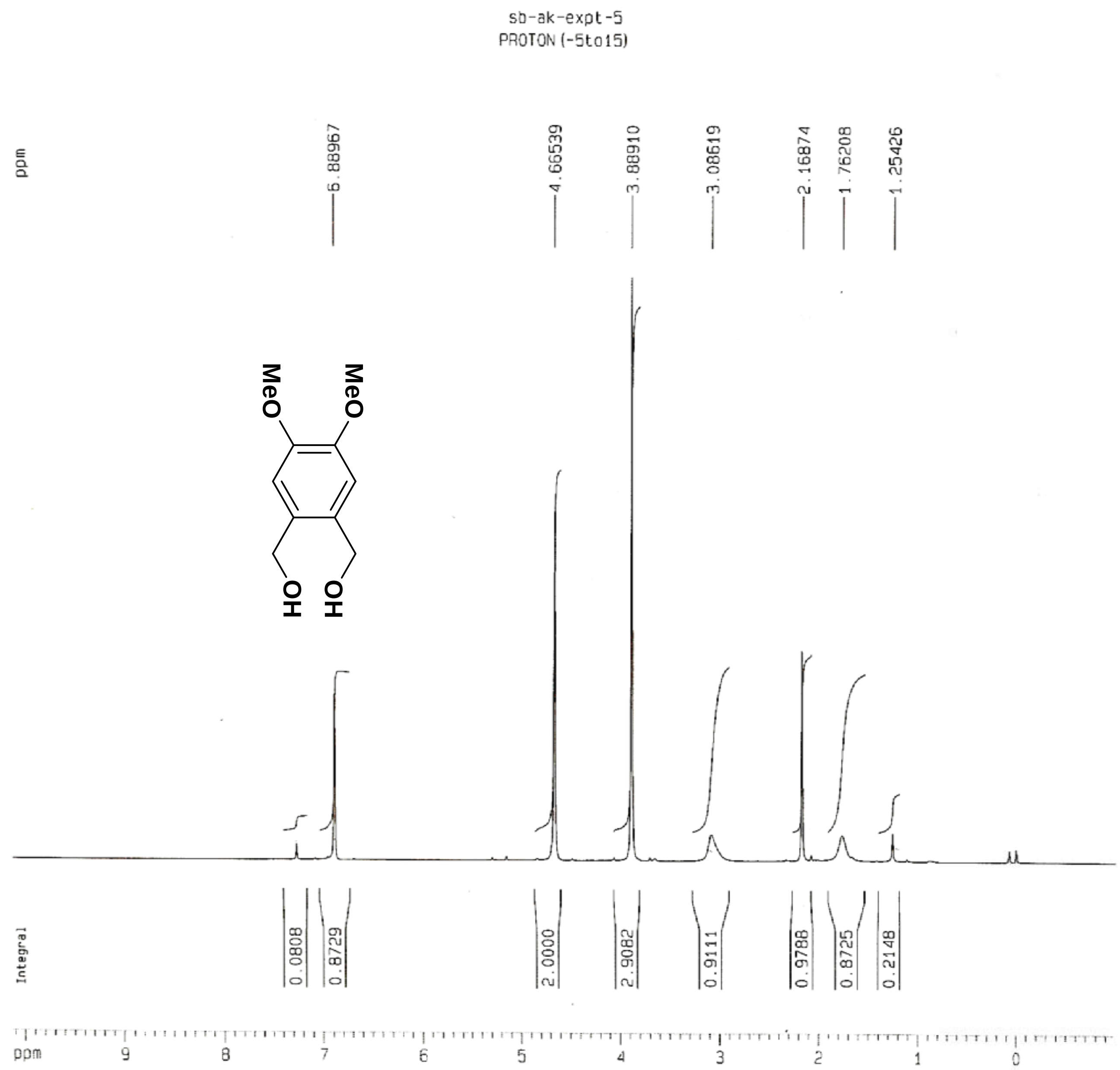


```
Current Data Parameters
NAME                sbak
EXPNO                  4
PROCNO                 1

F2 - Acquisition Parameters
Date_           20050608
Time               13.16
INSTRUM            av400
PROBHD   5 mm BBO BB-1H
PULPROG               zg
TD                 32768
SOLVENT            CDCl3
NS                    16
DS                     0
SWH             8389.262 Hz
FIDRES          0.256020 Hz
AQ             1.9530228 sec
RG                    64
DW                59.600 usec
DE                  6.00 usec
TE                 300.0 K
D1            2.00000000 sec

======== CHANNEL f1 ========
NUC1                  1H
P1                  8.35 usec
PL1                 0.00 dB
SFO1         400.1319460 MHz

F2 - Processing parameters
SI                 16384
SF           400.1300049 MHz
WDW                   EM
SSB                    0
LB                  0.30 Hz
GB                     0
PC                  3.00

1D NMR plot parameters
CX                 20.00 cm
CY                 10.00 cm
F1P               10.129 ppm
F1               4052.73 Hz
F2P               -1.000 ppm
F2               -400.13 Hz
PPMCM            0.55643 ppm/cm
HZCM           222.64290 Hz/cm
```

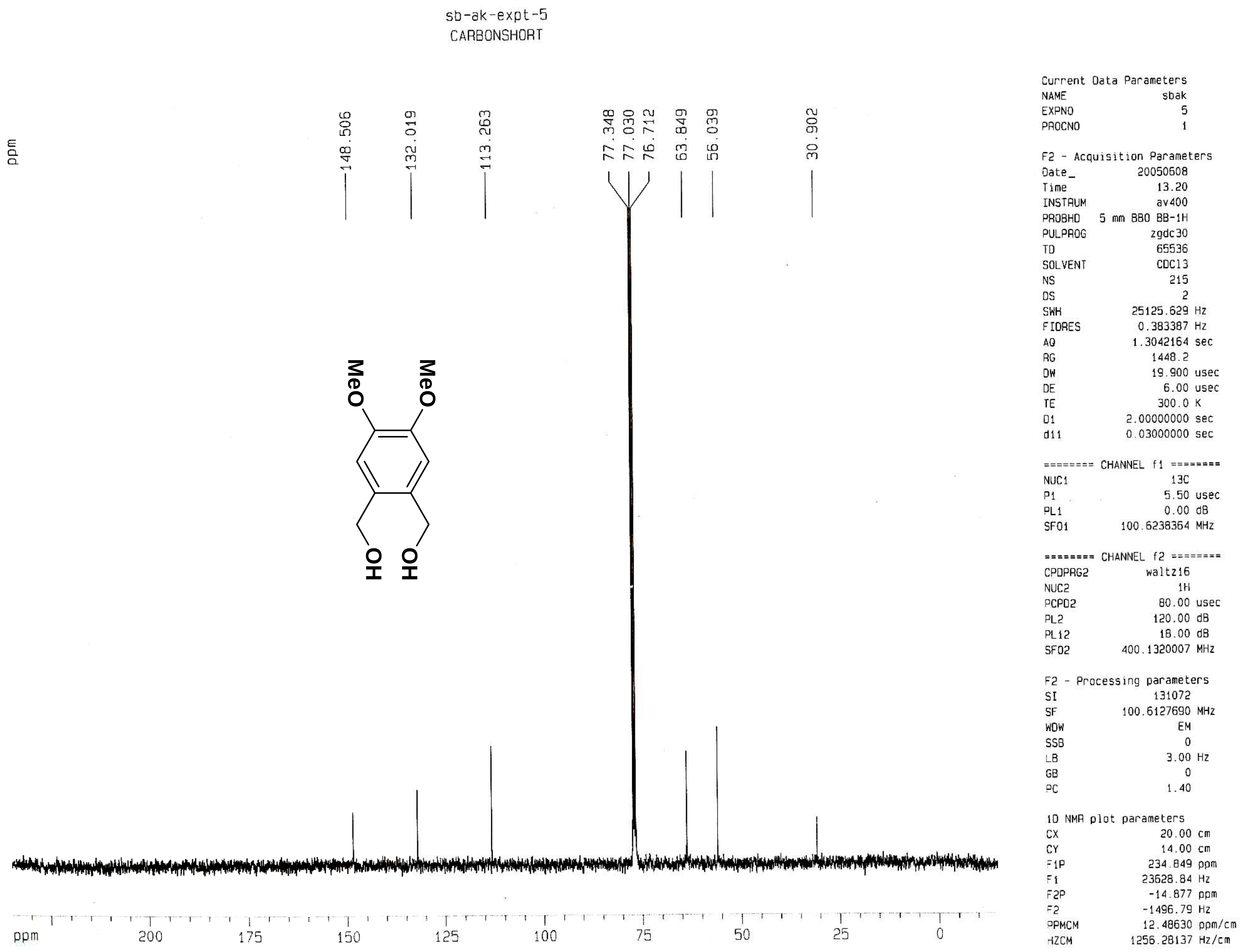
sb-ak-expt-5
CARBONSHORT
148.506
132.019
113.263
77.348
77.030
76.712
63.849
56.039
30.902
MeO
MeO
OH
OH
ppm
200
175
150
125
100
75
50
25
0
Current Data Parameters
NAME sbak
EXPNO 5
PROCNO 1
F2 - Acquisition Parameters
Date_ 20050608
Time 13.20
INSTRUM av400
PROBHD 5 mm BBO BB-1H
PULPROG zgdc30
TD 65536
SOLVENT CDCl3
NS 215
DS 2
SWH 25125.629 Hz
FIDRES 0.383387 Hz
AQ 1.3042164 sec
RG 1448.2
DW 19.900 usec
DE 6.00 usec
TE 300.0 K
D1 2.00000000 sec
d11 0.03000000 sec
======== CHANNEL f1 ========
NUC1 13C
P1 5.50 usec
PL1 0.00 dB
SFO1 100.6238364 MHz
======== CHANNEL f2 ========
CPDPRG2 waltz16
NUC2 1H
PCPD2 80.00 usec
PL2 120.00 dB
PL12 18.00 dB
SFO2 400.1320007 MHz
F2 - Processing parameters
SI 131072
SF 100.6127690 MHz
WDW EM
SSB 0
LB 3.00 Hz
GB 0
PC 1.40
1D NMR plot parameters
CX 20.00 cm
CY 14.00 cm
F1P 234.849 ppm
F1 23628.84 Hz
F2P -14.877 ppm
F2 -1496.79 Hz
PPMCM 12.48630 ppm/cm
HZCM 1256.28137 Hz/cm

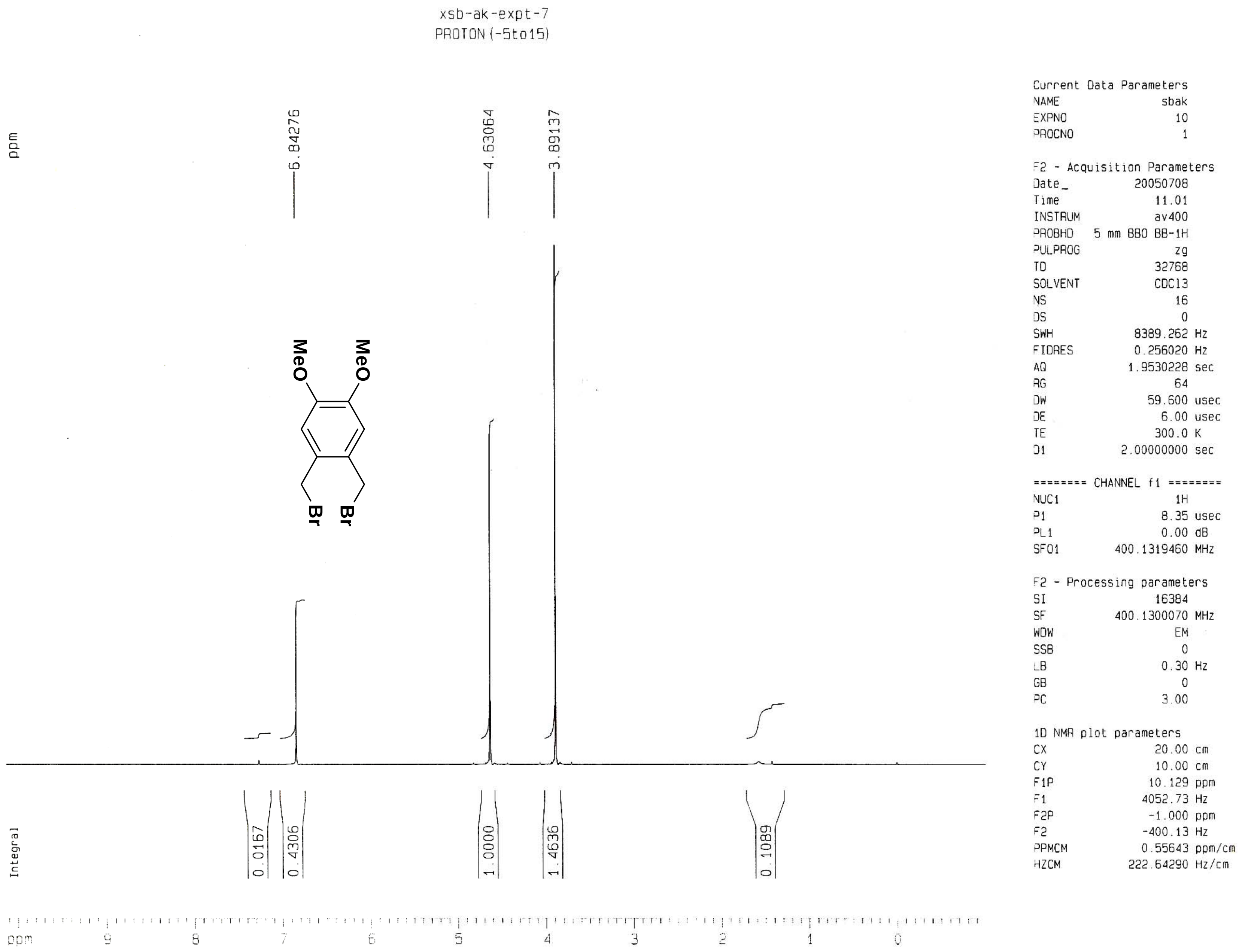
xsb-ak-expt-7
PROTON (-5to15)
6.84276
4.63064
3.89137
MeO
MeO
Br
Br
0.0167
0.4306
1.0000
1.4636
0.1089
Integral
ppm
Current Data Parameters
NAME sbak
EXPNO 10
PROCNO 1
F2 - Acquisition Parameters
Date_ 20050708
Time 11.01
INSTRUM av400
PROBHD 5 mm BBO BB-1H
PULPROG zg
TD 32768
SOLVENT CDCl3
NS 16
DS 0
SWH 8389.262 Hz
FIDRES 0.256020 Hz
AQ 1.9530228 sec
RG 64
DW 59.600 usec
DE 6.00 usec
TE 300.0 K
D1 2.00000000 sec
======== CHANNEL f1 ========
NUC1 1H
P1 8.35 usec
PL1 0.00 dB
SFO1 400.1319460 MHz
F2 - Processing parameters
SI 16384
SF 400.1300070 MHz
WDW EM
SSB 0
LB 0.30 Hz
GB 0
PC 3.00
1D NMR plot parameters
CX 20.00 cm
CY 10.00 cm
F1P 10.129 ppm
F1 4052.73 Hz
F2P -1.000 ppm
F2 -400.13 Hz
PPMCM 0.55643 ppm/cm
HZCM 222.64290 Hz/cm
ppm 9 8 7 6 5 4 3 2 1 0

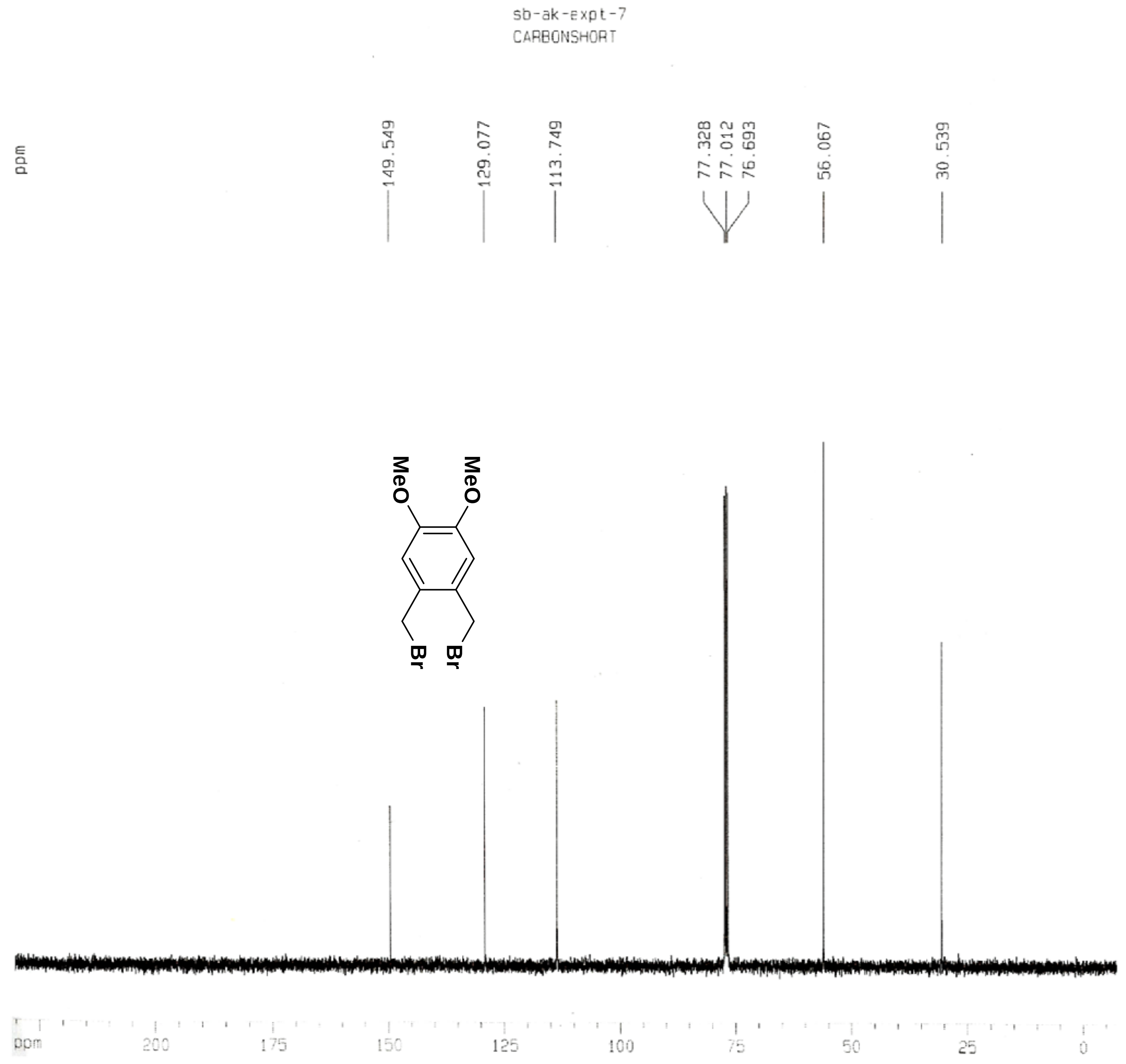

Current Data Parameters
NAME sbak
EXPNO 11
PROCNO 1

F2 - Acquisition Parameters
Date_ 20050708
Time 10.42
INSTRUM av400
PROBHD 5 mm BBO BB-1H
PULPROG zgdc30
TD 65536
SOLVENT CDCl3
NS 363
DS 2
SWH 25125.629 Hz
FIDRES 0.383387 Hz
AQ 1.3042164 sec
RG 181
DW 19.900 usec
DE 6.00 usec
TE 300.0 K
D1 2.00000000 sec
d11 0.03000000 sec

======== CHANNEL f1 ========
NUC1 13C
P1 5.50 usec
PL1 0.00 dB
SFO1 100.6238364 MHz

======== CHANNEL f2 ========
CPDPRG2 waltz16
NUC2 1H
PCPD2 80.00 usec
PL2 120.00 dB
PL12 18.00 dB
SFO2 400.1320007 MHz

F2 - Processing parameters
SI 131072
SF 100.6127690 MHz
WDW EM
SSB 0
LB 1.00 Hz
GB 0
PC 1.40

1D NMR plot parameters
CX 20.00 cm
CY 9.10 cm
F1P 230.605 ppm
F1 23201.76 Hz
F2P -7.259 ppm
F2 -730.32 Hz
PPMCM 11.89316 ppm/cm
HZCM 1196.60425 Hz/cm

SB/AK/10
PROTON(-5to15)

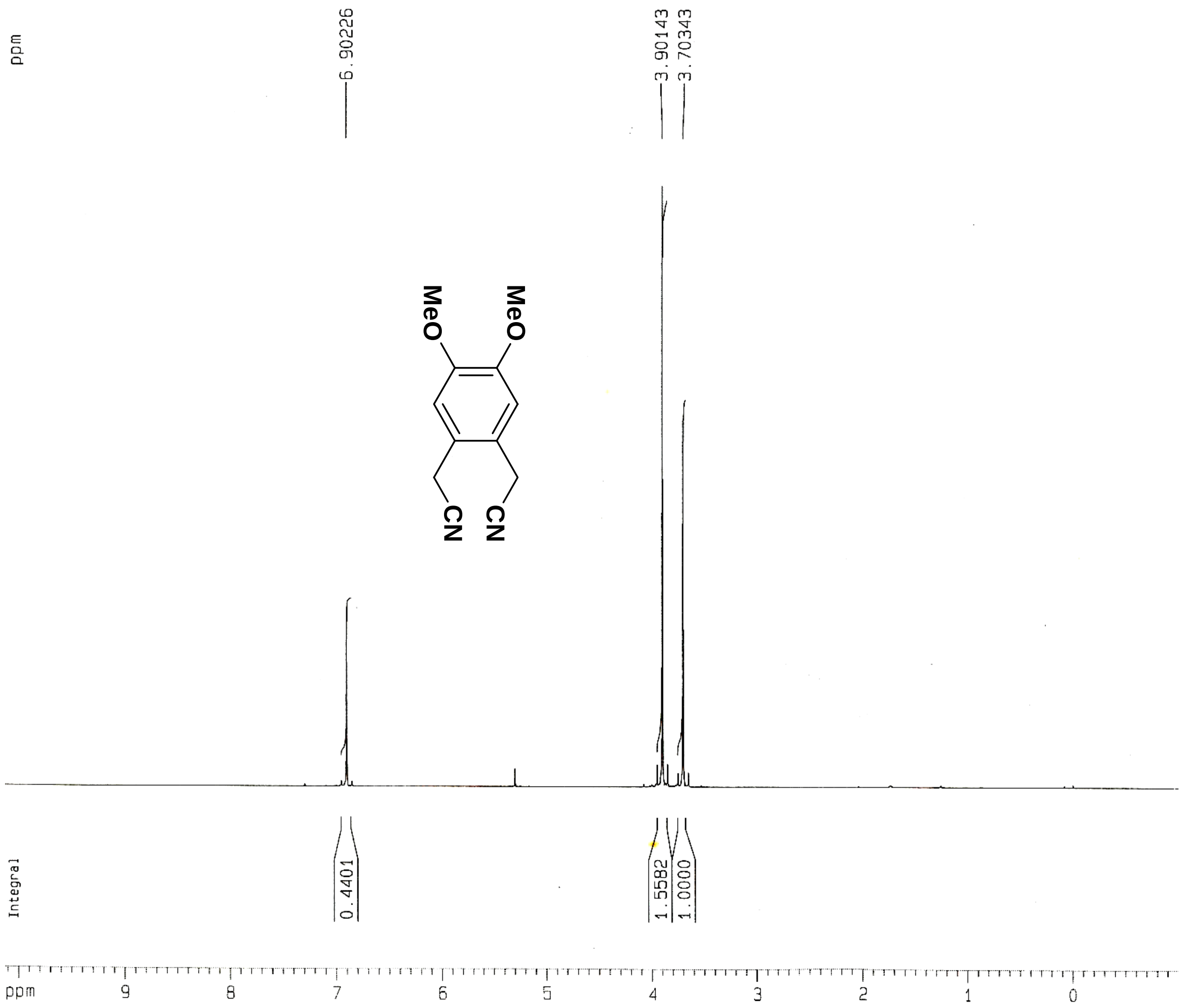


```
Current Data Parameters
NAME              sbak
EXPNO               18
PROCNO               1

F2 - Acquisition Parameters
Date_         20050722
Time             17.25
INSTRUM          av400
PROBHD   5 mm BBO BB-1H
PULPROG             zg
TD               32768
SOLVENT          CDCl3
NS                  16
DS                   0
SWH           8389.262 Hz
FIDRES        0.256020 Hz
AQ           1.9530228 sec
RG                22.6
DW              59.600 usec
DE                6.00 usec
TE               300.0 K
D1          2.00000000 sec

======== CHANNEL f1 ========
NUC1                1H
P1                8.35 usec
PL1               0.00 dB
SFO1       400.1319460 MHz

F2 - Processing parameters
SI               16384
SF         400.1299951 MHz
WDW                 EM
SSB                  0
LB                0.30 Hz
GB                   0
PC                3.00

1D NMR plot parameters
CX               20.00 cm
CY               10.00 cm
F1P             10.129 ppm
F1             4052.73 Hz
F2P             -1.000 ppm
F2             -400.13 Hz
PPMCM          0.55643 ppm/cm
HZCM         222.64290 Hz/cm
```

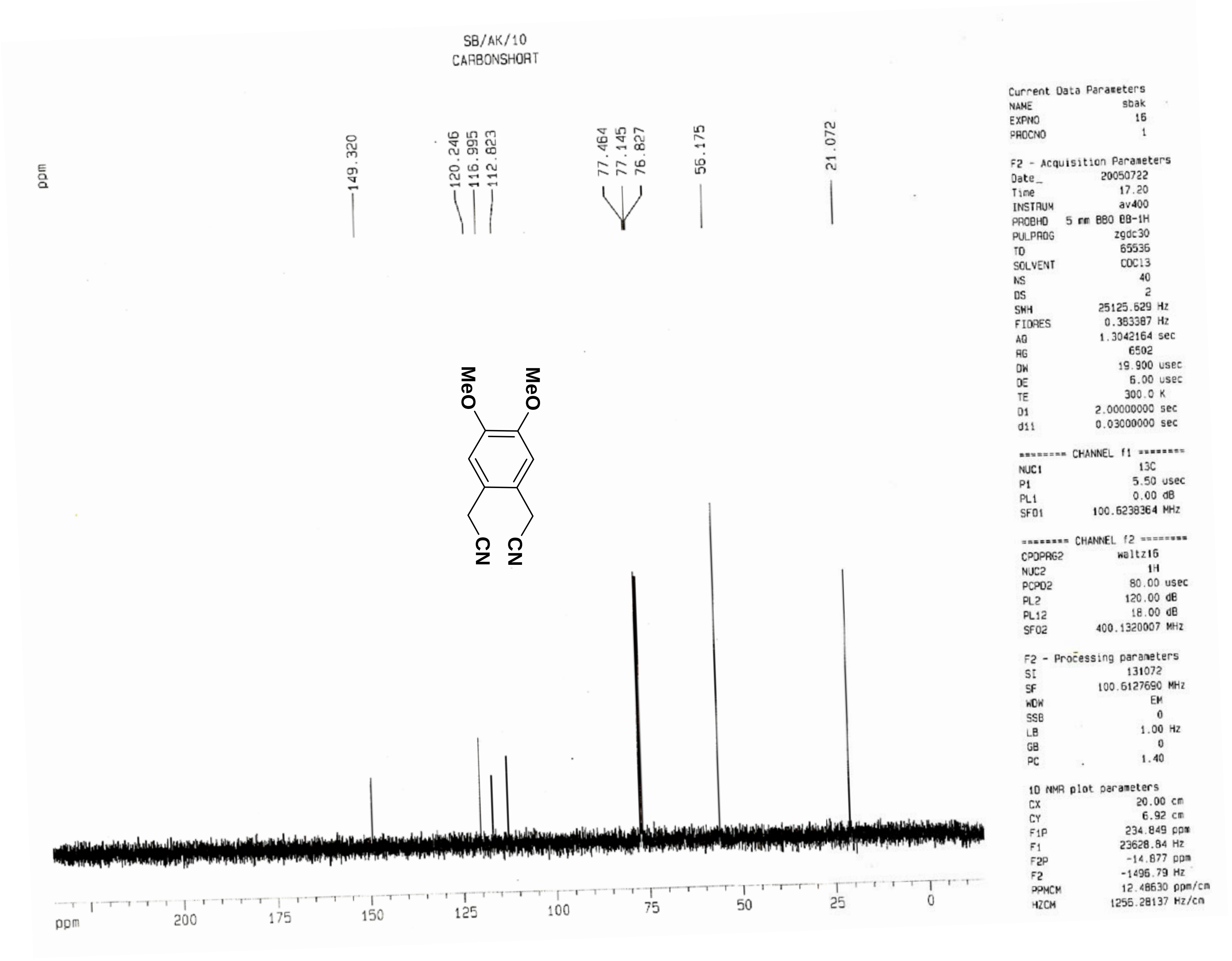
SB/AK/10
CARBONSHORT
149.320
120.246
116.995
112.823
77.464
77.145
76.827
56.175
21.072
ppm
MeO
MeO
CN
CN
Current Data Parameters
NAME sbak
EXPNO 16
PROCNO 1
F2 - Acquisition Parameters
Date_ 20050722
Time 17.20
INSTRUM av400
PROBHD 5 mm BBO BB-1H
PULPROG zgdc30
TD 65536
SOLVENT CDCl3
NS 40
DS 2
SWH 25125.629 Hz
FIDRES 0.383387 Hz
AQ 1.3042164 sec
RG 6502
DW 19.900 usec
DE 6.00 usec
TE 300.0 K
D1 2.00000000 sec
d11 0.03000000 sec
======== CHANNEL f1 ========
NUC1 13C
P1 5.50 usec
PL1 0.00 dB
SFO1 100.6238364 MHz
======== CHANNEL f2 ========
CPDPRG2 waltz16
NUC2 1H
PCPD2 80.00 usec
PL2 120.00 dB
PL12 18.00 dB
SFO2 400.1320007 MHz
F2 - Processing parameters
SI 131072
SF 100.6127690 MHz
WDW EM
SSB 0
LB 1.00 Hz
GB 0
PC 1.40
1D NMR plot parameters
CX 20.00 cm
CY 6.92 cm
F1P 234.849 ppm
F1 23628.84 Hz
F2P -14.877 ppm
F2 -1496.79 Hz
PPMCM 12.48630 ppm/cm
HZCM 1256.28137 Hz/cm
ppm
200
175
150
125
100
75
50
25
0

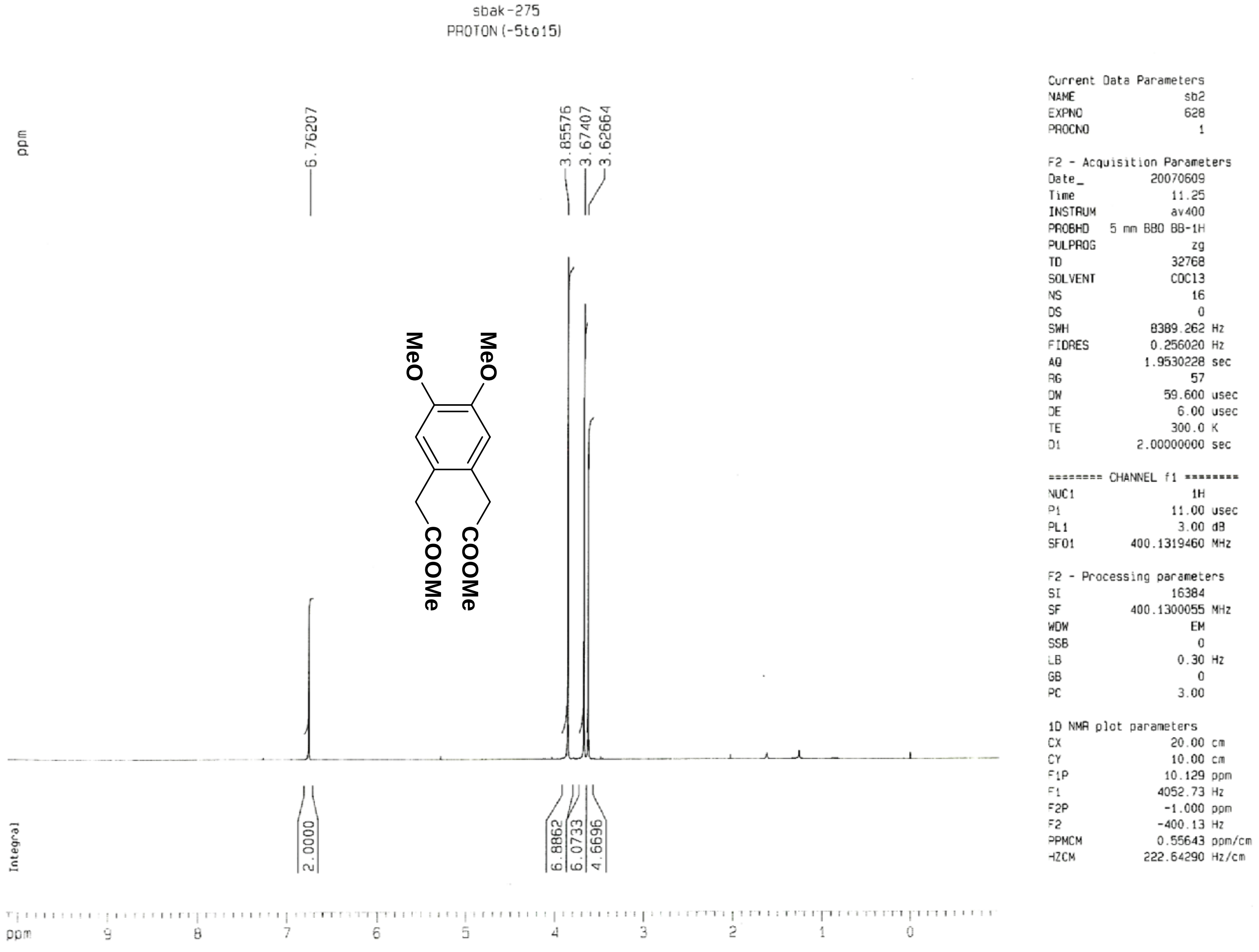
sbak-275
PROTON (-5to15)
6.76207
3.85576
3.67407
3.62664
MeO
MeO
COOMe
COOMe
2.0000
6.8862
6.0733
4.6696
Integral
ppm
9
8
7
6
5
4
3
2
1
0
Current Data Parameters
NAME sb2
EXPNO 628
PROCNO 1
F2 - Acquisition Parameters
Date_ 20070609
Time 11.25
INSTRUM av400
PROBHD 5 mm BBO BB-1H
PULPROG zg
TD 32768
SOLVENT CDCl3
NS 16
DS 0
SWH 8389.262 Hz
FIDRES 0.256020 Hz
AQ 1.9530228 sec
RG 57
DW 59.600 usec
DE 6.00 usec
TE 300.0 K
D1 2.00000000 sec
======== CHANNEL f1 ========
NUC1 1H
P1 11.00 usec
PL1 3.00 dB
SFO1 400.1319460 MHz
F2 - Processing parameters
SI 16384
SF 400.1300055 MHz
WDW EM
SSB 0
LB 0.30 Hz
GB 0
PC 3.00
1D NMR plot parameters
CX 20.00 cm
CY 10.00 cm
F1P 10.129 ppm
F1 4052.73 Hz
F2P -1.000 ppm
F2 -400.13 Hz
PPMCM 0.55643 ppm/cm
HZCM 222.64290 Hz/cm

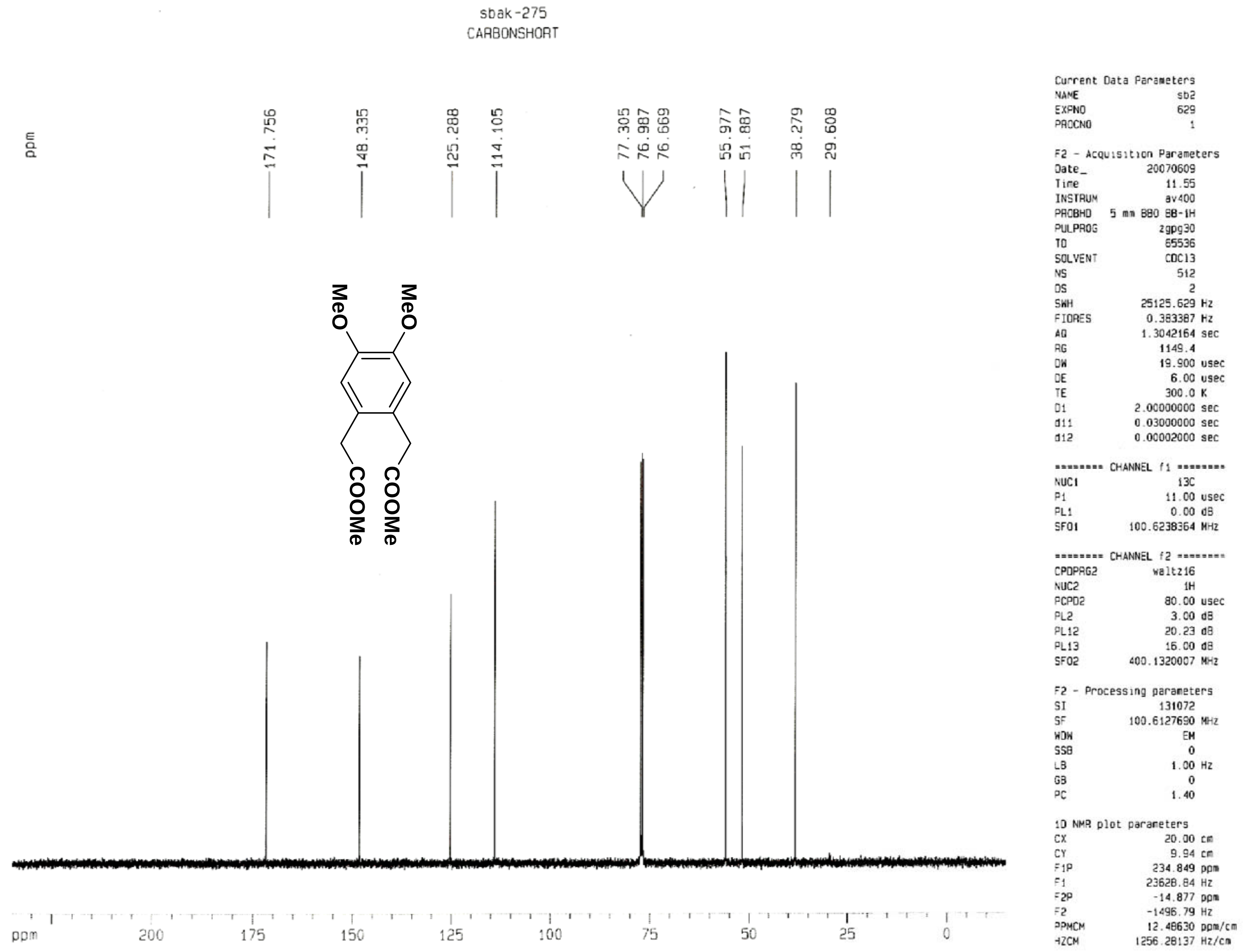
sbak-275
CARBONSHORT
ppm
171.756
148.335
125.288
114.105
77.305
76.987
76.669
55.977
51.887
38.279
29.608
MeO
MeO
COOMe
COOMe
ppm 200 175 150 125 100 75 50 25 0
Current Data Parameters
NAME sb2
EXPNO 629
PROCNO 1
F2 - Acquisition Parameters
Date_ 20070609
Time 11.55
INSTRUM av400
PROBHD 5 mm BBO BB-1H
PULPROG zgpg30
TD 65536
SOLVENT CDCl3
NS 512
DS 2
SWH 25125.629 Hz
FIDRES 0.383387 Hz
AQ 1.3042164 sec
RG 1149.4
DW 19.900 usec
DE 6.00 usec
TE 300.0 K
D1 2.00000000 sec
d11 0.03000000 sec
d12 0.00002000 sec
======== CHANNEL f1 ========
NUC1 13C
P1 11.00 usec
PL1 0.00 dB
SFO1 100.6238364 MHz
======== CHANNEL f2 ========
CPDPRG2 waltz16
NUC2 1H
PCPD2 80.00 usec
PL2 3.00 dB
PL12 20.23 dB
PL13 16.00 dB
SFO2 400.1320007 MHz
F2 - Processing parameters
SI 131072
SF 100.6127690 MHz
WDW EM
SSB 0
LB 1.00 Hz
GB 0
PC 1.40
1D NMR plot parameters
CX 20.00 cm
CY 9.94 cm
F1P 234.849 ppm
F1 23628.84 Hz
F2P -14.877 ppm
F2 -1496.79 Hz
PPMCM 12.48630 ppm/cm
HZCM 1256.28137 Hz/cm

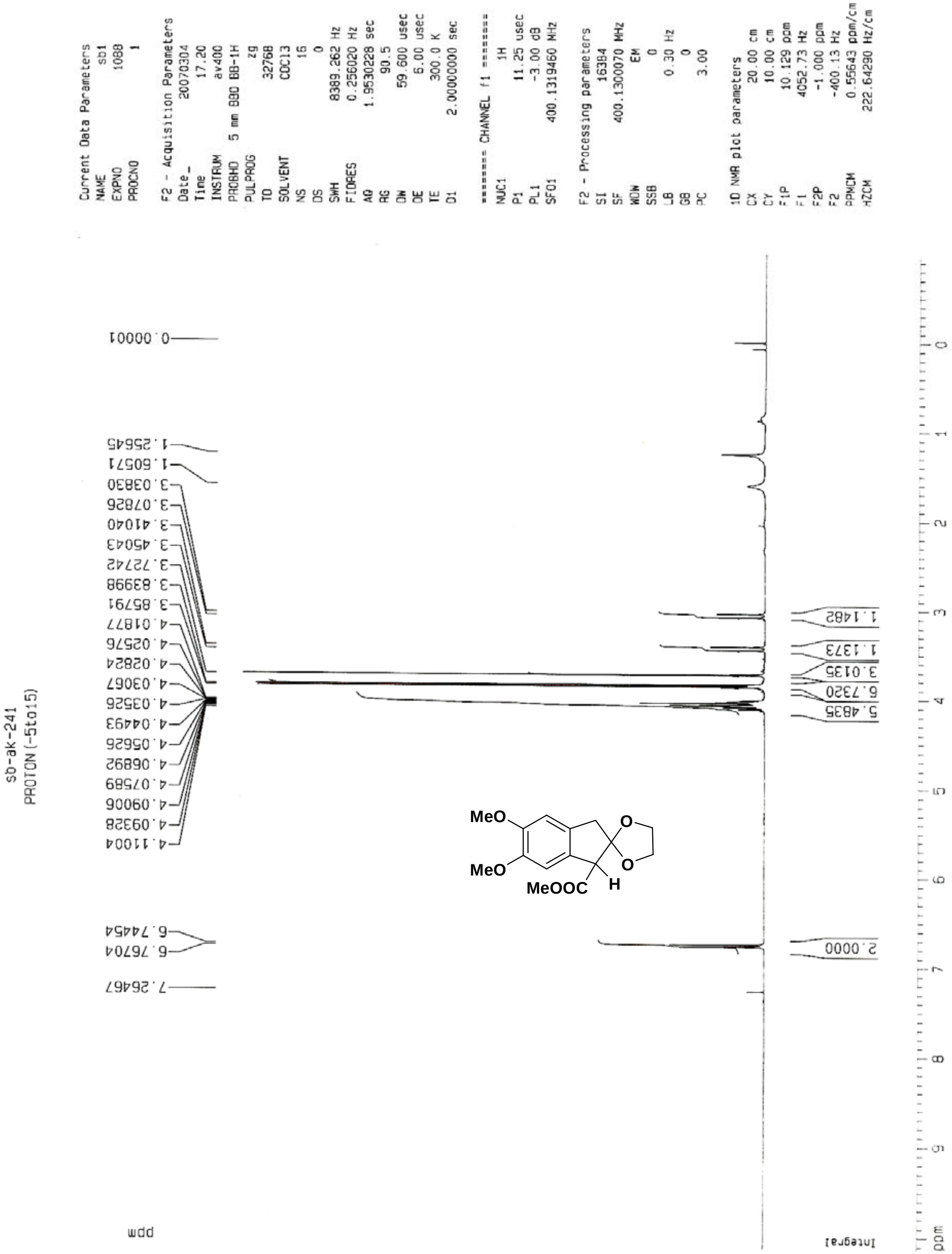
sb-ak-241
PROTON (-5to15)
Current Data Parameters
NAME sb1
EXPNO 1088
PROCNO 1
F2 - Acquisition Parameters
Date_ 20070304
Time 17.20
INSTRUM av400
PROBHD 5 mm BBO BB-1H
PULPROG zg
TD 32768
SOLVENT CDCl3
NS 16
DS 0
SWH 8389.262 Hz
FIDRES 0.256020 Hz
AQ 1.9530228 sec
RG 90.5
DW 59.600 usec
DE 6.00 usec
TE 300.0 K
D1 2.00000000 sec
======== CHANNEL f1 ========
NUC1 1H
P1 11.25 usec
PL1 -3.00 dB
SFO1 400.1319460 MHz
F2 - Processing parameters
SI 16384
SF 400.1300070 MHz
WDW EM
SSB 0
LB 0.30 Hz
GB 0
PC 3.00
1D NMR plot parameters
CX 20.00 cm
CY 10.00 cm
F1P 10.129 ppm
F1 4052.73 Hz
F2P -1.000 ppm
F2 -400.13 Hz
PPMCM 0.55643 ppm/cm
HZCM 222.64290 Hz/cm
-0.00001
1.25645
1.60571
3.03830
3.07826
3.41040
3.45043
3.72742
3.83998
3.85791
4.01877
4.02576
4.02824
4.03067
4.03526
4.04493
4.05626
4.06892
4.07589
4.09006
4.09328
4.11004
6.74454
6.76704
7.26467
1.1482
1.1373
3.0135
6.7320
5.4835
2.0000
Integral
ppm
MeO
MeO
MeOOC
H
O
O

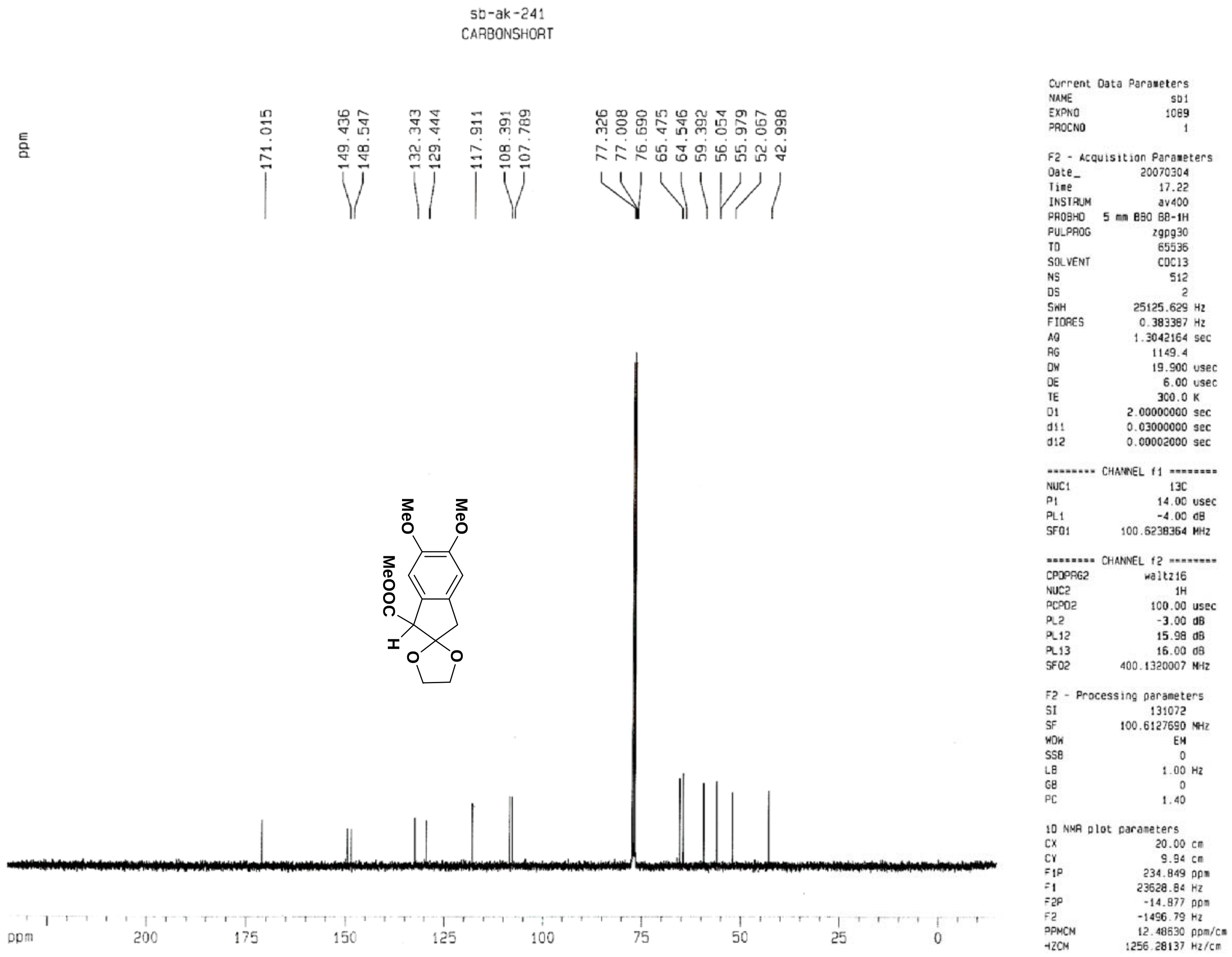
sb-ak-241
CARBONSHORT
171.015
149.436
148.547
132.343
129.444
117.911
108.391
107.789
77.326
77.008
76.690
65.475
64.546
59.392
56.054
55.979
52.067
42.998
MeO
MeO
MeOOC
H
O
O
ppm
200
175
150
125
100
75
50
25
0
Current Data Parameters
NAME sb1
EXPNO 1089
PROCNO 1
F2 - Acquisition Parameters
Date_ 20070304
Time 17.22
INSTRUM av400
PROBHD 5 mm BBO BB-1H
PULPROG zgpg30
TD 65536
SOLVENT CDCl3
NS 512
DS 2
SWH 25125.629 Hz
FIDRES 0.383387 Hz
AQ 1.3042164 sec
RG 1149.4
DW 19.900 usec
DE 6.00 usec
TE 300.0 K
D1 2.00000000 sec
d11 0.03000000 sec
d12 0.00002000 sec
======== CHANNEL f1 ========
NUC1 13C
P1 14.00 usec
PL1 -4.00 dB
SFO1 100.6238364 MHz
======== CHANNEL f2 ========
CPDPRG2 waltz16
NUC2 1H
PCPD2 100.00 usec
PL2 -3.00 dB
PL12 15.98 dB
PL13 16.00 dB
SFO2 400.1320007 MHz
F2 - Processing parameters
SI 131072
SF 100.6127690 MHz
WDW EM
SSB 0
LB 1.00 Hz
GB 0
PC 1.40
1D NMR plot parameters
CX 20.00 cm
CY 9.94 cm
F1P 234.849 ppm
F1 23628.84 Hz
F2P -14.877 ppm
F2 -1496.79 Hz
PPMCM 12.48630 ppm/cm
HZCM 1256.28137 Hz/cm

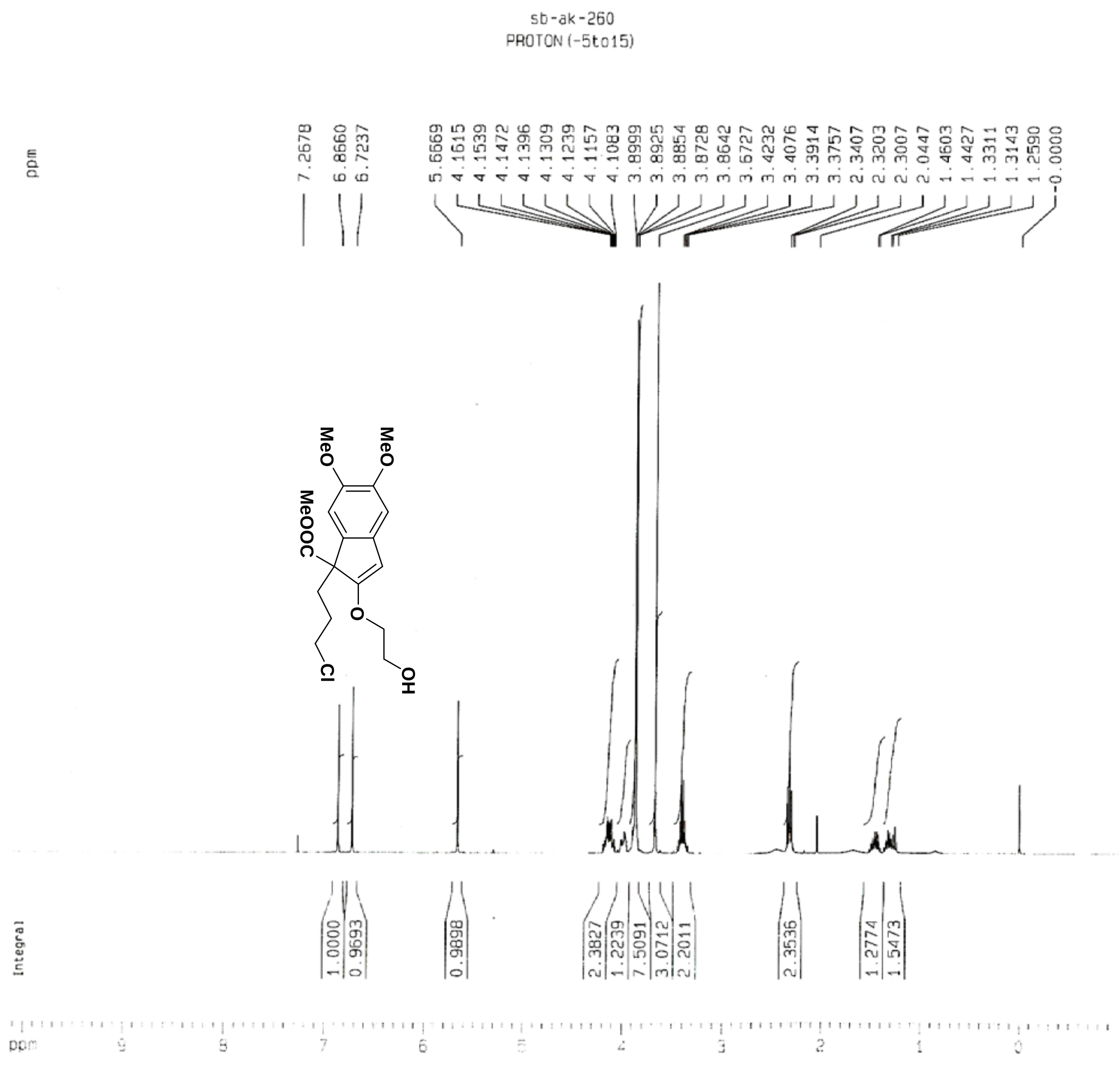

Current Data Parameters
NAME sb2
EXPNO 122
PROCNO 1

F2 - Acquisition Parameters
Date_ 20070408
Time 16.26
INSTRUM av400
PROBHD 5 mm BBO BB-1H
PULPROG zg
TD 32768
SOLVENT CDCl3
NS 16
DS 0
SWH 8389.262 Hz
FIDRES 0.256020 Hz
AQ 1.9530228 sec
RG 64
DW 59.600 usec
DE 6.00 usec
TE 300.0 K
D1 2.00000000 sec

======== CHANNEL f1 ========
NUC1 1H
P1 11.00 usec
PL1 3.00 dB
SFO1 400.1319460 MHz

F2 - Processing parameters
SI 16384
SF 400.1300058 MHz
WDW EM
SSB 0
LB 0.30 Hz
GB 0
PC 3.00

1D NMR plot parameters
CX 20.00 cm
CY 10.00 cm
F1P 10.129 ppm
F1 4052.73 Hz
F2P -1.000 ppm
F2 -400.13 Hz
PPMCM 0.55643 ppm/cm
HZCM 222.64290 Hz/cm

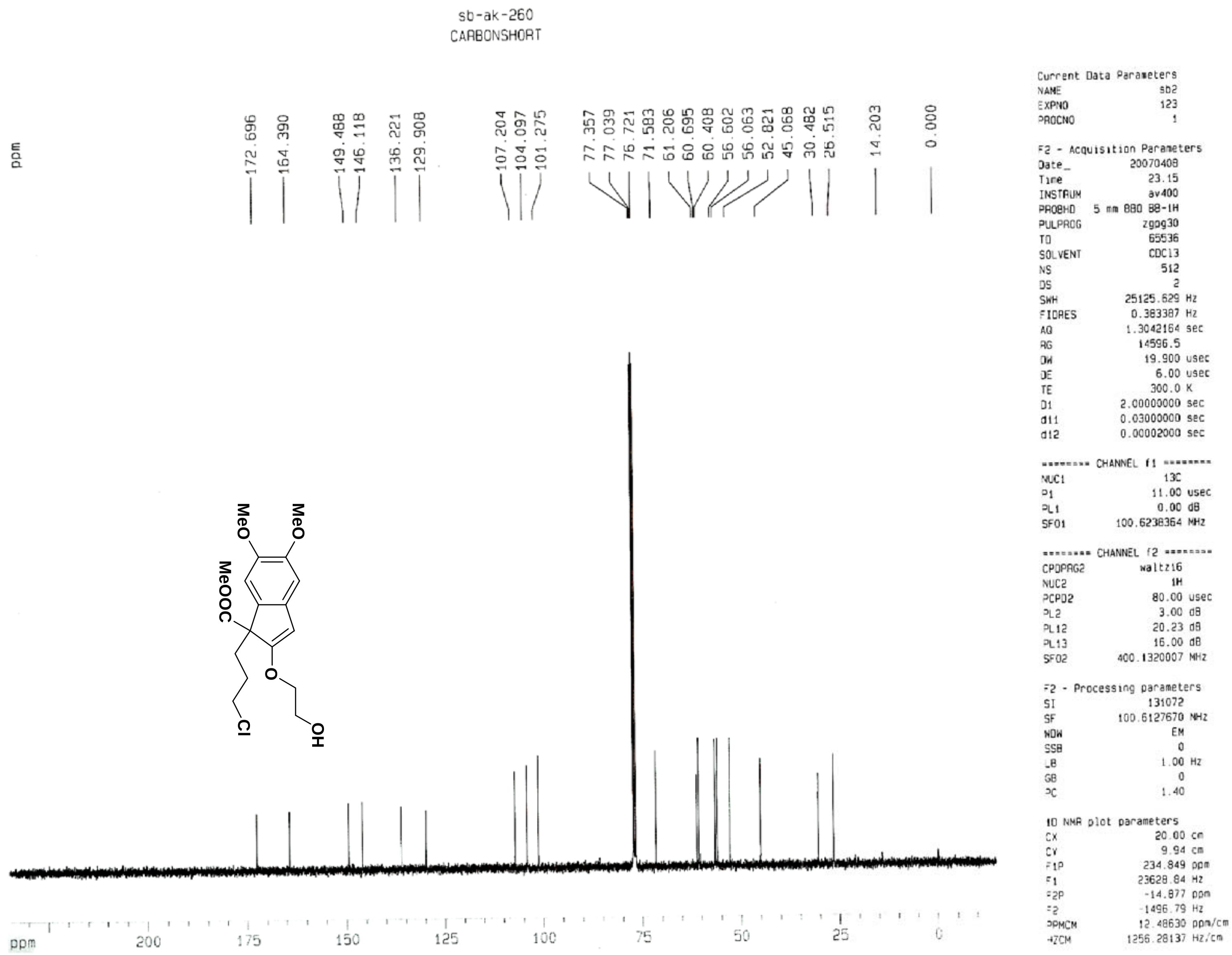

sb-ak-260
CARBONSHORT
172.696
164.390
149.488
146.118
136.221
129.908
107.204
104.097
101.275
77.357
77.039
76.721
71.583
61.206
60.695
60.408
56.602
56.063
52.821
45.068
30.482
26.515
14.203
0.000
ppm
MeO
MeO
MeOOC
O
OH
Cl
ppm 200 175 150 125 100 75 50 25 0
Current Data Parameters
NAME sb2
EXPNO 123
PROCNO 1
F2 - Acquisition Parameters
Date_ 20070408
Time 23.15
INSTRUM av400
PROBHD 5 mm BBO BB-1H
PULPROG zgpg30
TD 65536
SOLVENT CDCl3
NS 512
DS 2
SWH 25125.629 Hz
FIDRES 0.383387 Hz
AQ 1.3042164 sec
RG 14596.5
DW 19.900 usec
DE 6.00 usec
TE 300.0 K
D1 2.00000000 sec
d11 0.03000000 sec
d12 0.00002000 sec
======== CHANNEL f1 ========
NUC1 13C
P1 11.00 usec
PL1 0.00 dB
SFO1 100.6238364 MHz
======== CHANNEL f2 ========
CPDPRG2 waltz16
NUC2 1H
PCPD2 80.00 usec
PL2 3.00 dB
PL12 20.23 dB
PL13 16.00 dB
SFO2 400.1320007 MHz
F2 - Processing parameters
SI 131072
SF 100.6127670 MHz
WDW EM
SSB 0
LB 1.00 Hz
GB 0
PC 1.40
1D NMR plot parameters
CX 20.00 cm
CY 9.94 cm
F1P 234.849 ppm
F1 23628.84 Hz
F2P -14.877 ppm
F2 -1496.79 Hz
PPMCM 12.48630 ppm/cm
HZCM 1256.28137 Hz/cm

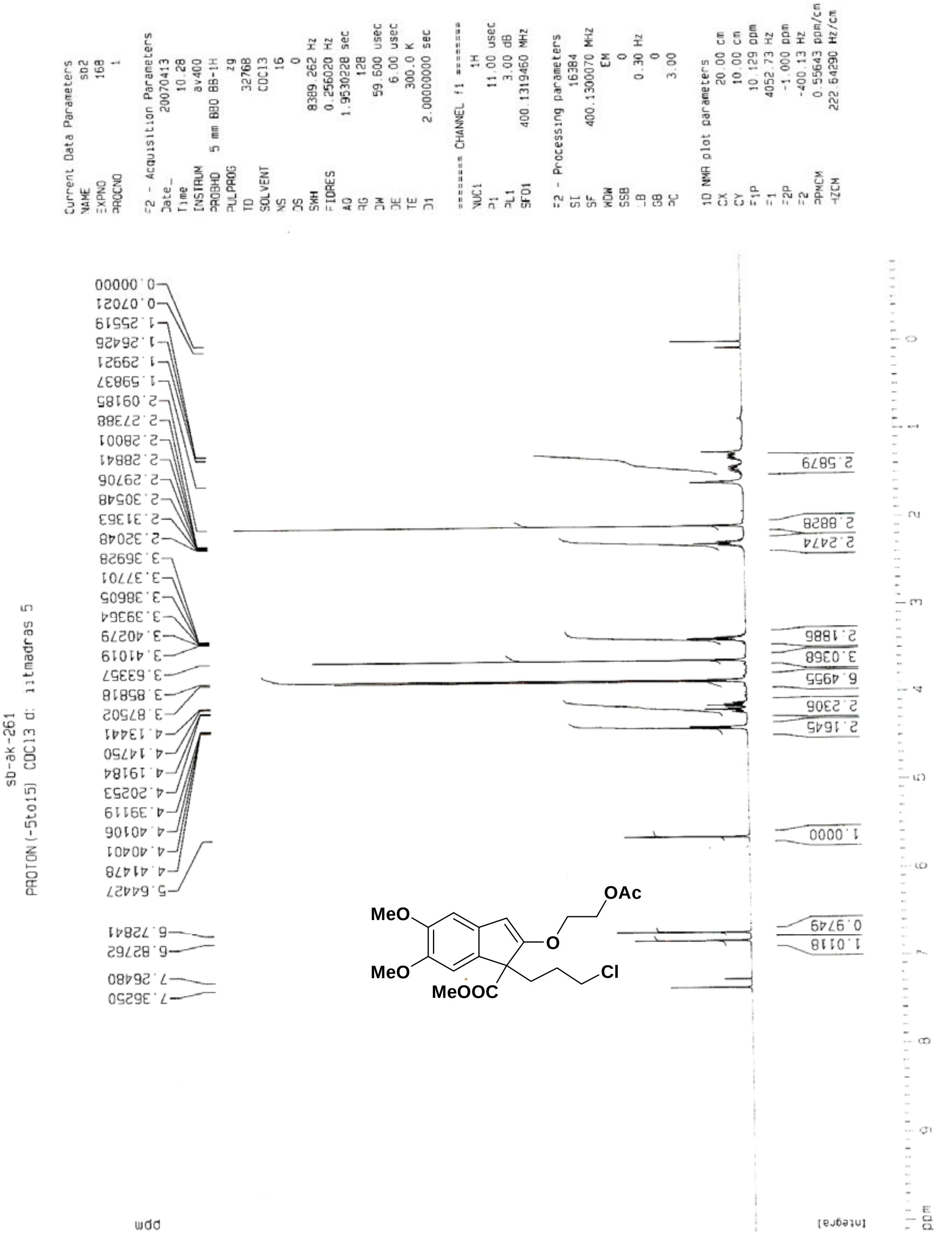

sb-ak-261
PROTON(-5to15) CDC13 d: iitmadras 5
Current Data Parameters
NAME sb2
EXPNO 168
PROCNO 1
F2 - Acquisition Parameters
Date_ 20070413
Time 10.28
INSTRUM av400
PROBHD 5 mm BBO BB-1H
PULPROG zg
TD 32768
SOLVENT CDC13
NS 16
DS 0
SWH 8389.262 Hz
FIDRES 0.256020 Hz
AQ 1.9530228 sec
RG 128
DW 59.600 usec
DE 6.00 usec
TE 300.0 K
D1 2.00000000 sec
======== CHANNEL f1 ========
NUC1 1H
P1 11.00 usec
PL1 3.00 dB
SFO1 400.1319460 MHz
F2 - Processing parameters
SI 16384
SF 400.1300070 MHz
WDW EM
SSB 0
LB 0.30 Hz
GB 0
PC 3.00
1D NMR plot parameters
CX 20.00 cm
CY 10.00 cm
F1P 10.129 ppm
F1 4052.73 Hz
F2P -1.000 ppm
F2 -400.13 Hz
PPMCM 0.55643 ppm/cm
HZCM 222.64290 Hz/cm
MeO
MeO
MeOOC
O
OAc
Cl
Integral
ppm

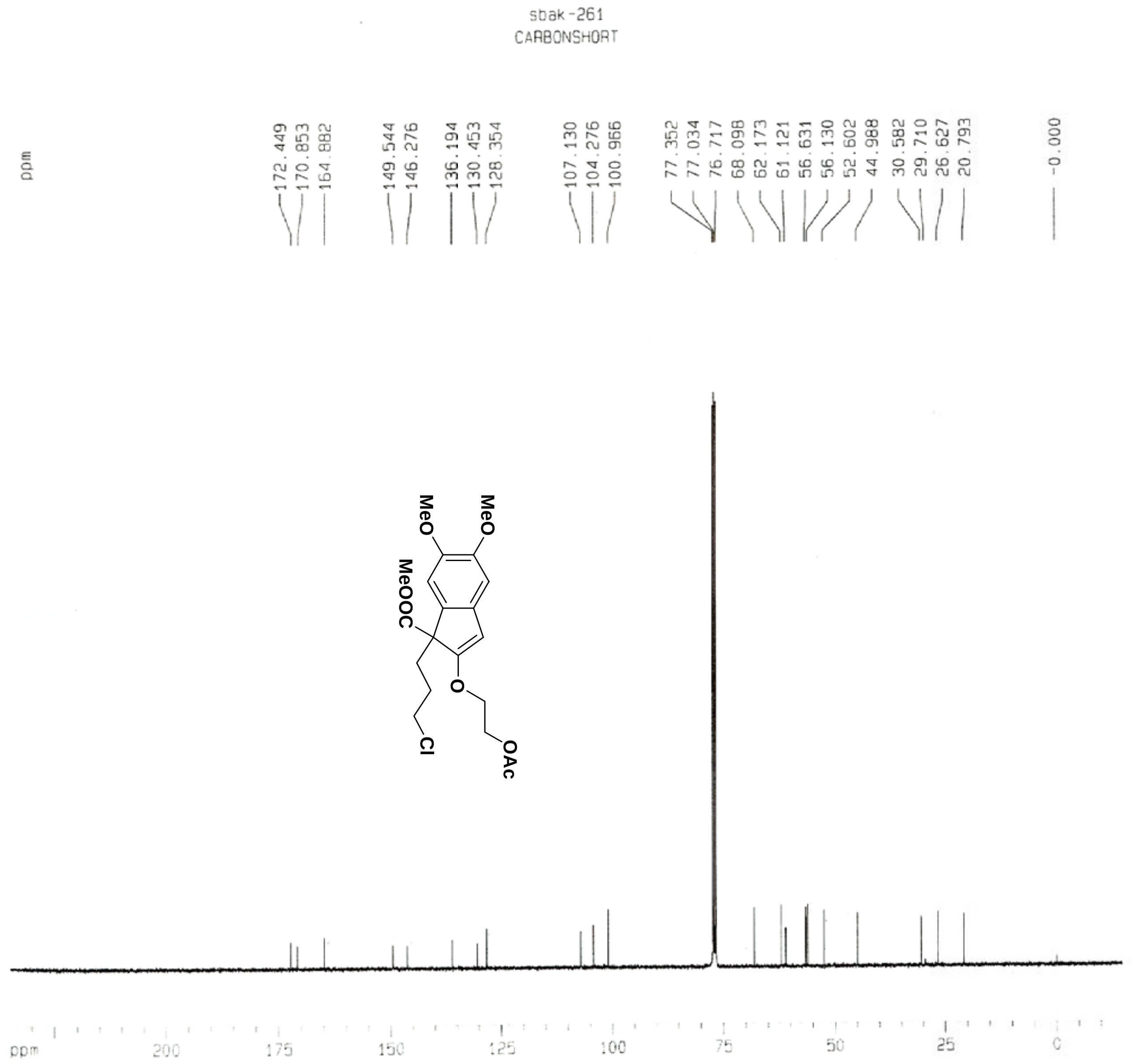

Current Data Parameters
NAME sb2
EXPNO 167
PROCNO 1

F2 - Acquisition Parameters
Date_ 20070413
Time 7.08
INSTRUM av400
PROBHD 5 mm BBO BB-1H
PULPROG zgpg30
TD 65536
SOLVENT CDCl3
NS 4000
DS 2
SWH 25125.629 Hz
FIDRES 0.383387 Hz
AQ 1.3042164 sec
RG 1149.4
DW 19.900 usec
DE 6.00 usec
TE 300.0 K
D1 2.00000000 sec
d11 0.03000000 sec
d12 0.00002000 sec

======== CHANNEL f1 ========
NUC1 13C
P1 11.00 usec
PL1 0.00 dB
SFO1 100.6238364 MHz

======== CHANNEL f2 ========
CPDPRG2 waltz16
NUC2 1H
PCPD2 80.00 usec
PL2 3.00 dB
PL12 20.23 dB
PL13 16.00 dB
SFO2 400.1320007 MHz

F2 - Processing parameters
SI 131072
SF 100.6127637 MHz
WDW EM
SSB 0
LB 1.00 Hz
GB 0
PC 1.40

1D NMR plot parameters
CX 20.00 cm
CY 9.94 cm
F1P 234.849 ppm
F1 23628.84 Hz
F2P -14.877 ppm
F2 -1496.79 Hz
PPMCM 12.48630 ppm/cm
HZCM 1256.28125 Hz/cm

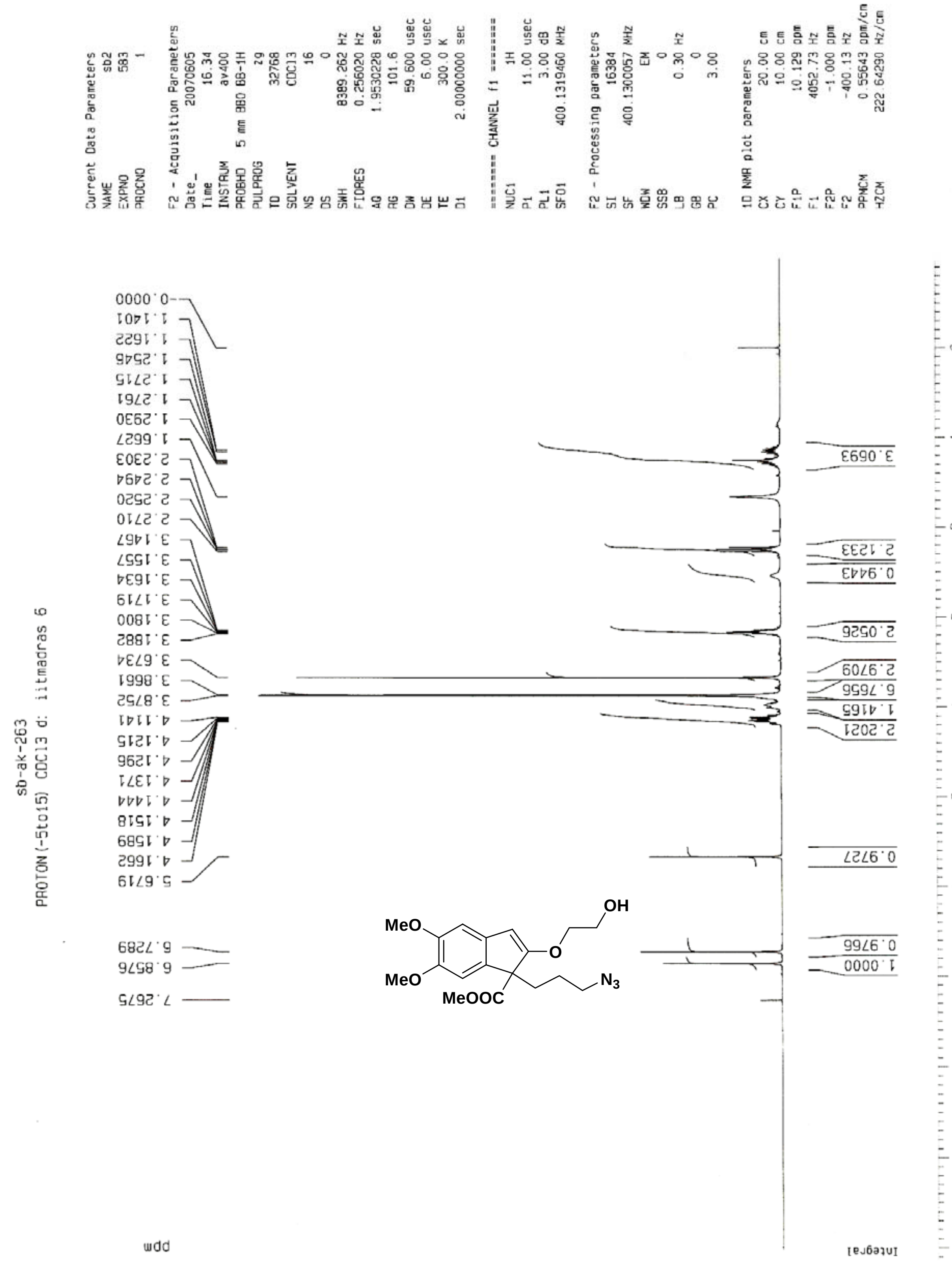
sb-ak-263
PROTON(-5to15) CDCl3 d: iitmadras 6
Current Data Parameters
NAME sb2
EXPNO 583
PROCNO 1
F2 - Acquisition Parameters
Date_ 20070605
Time 16.34
INSTRUM av400
PROBHD 5 mm BBO BB-1H
PULPROG zg
TD 32768
SOLVENT CDCl3
NS 16
DS 0
SWH 8389.262 Hz
FIDRES 0.256020 Hz
AQ 1.9530228 sec
RG 101.6
DW 59.600 usec
DE 6.00 usec
TE 300.0 K
D1 2.00000000 sec
======== CHANNEL f1 ========
NUC1 1H
P1 11.00 usec
PL1 3.00 dB
SFO1 400.1319460 MHz
F2 - Processing parameters
SI 16384
SF 400.1300057 MHz
WDW EM
SSB 0
LB 0.30 Hz
GB 0
PC 3.00
1D NMR plot parameters
CX 20.00 cm
CY 10.00 cm
F1P 10.129 ppm
F1 4052.73 Hz
F2P -1.000 ppm
F2 -400.13 Hz
PPMCM 0.55643 ppm/cm
HZCM 222.64290 Hz/cm
MeO
MeO
MeOOC
OH
O
N3
ppm
Integral
ppm

sb-ak-263
CARBONSHORT CDCl3 d: iitmadras 1

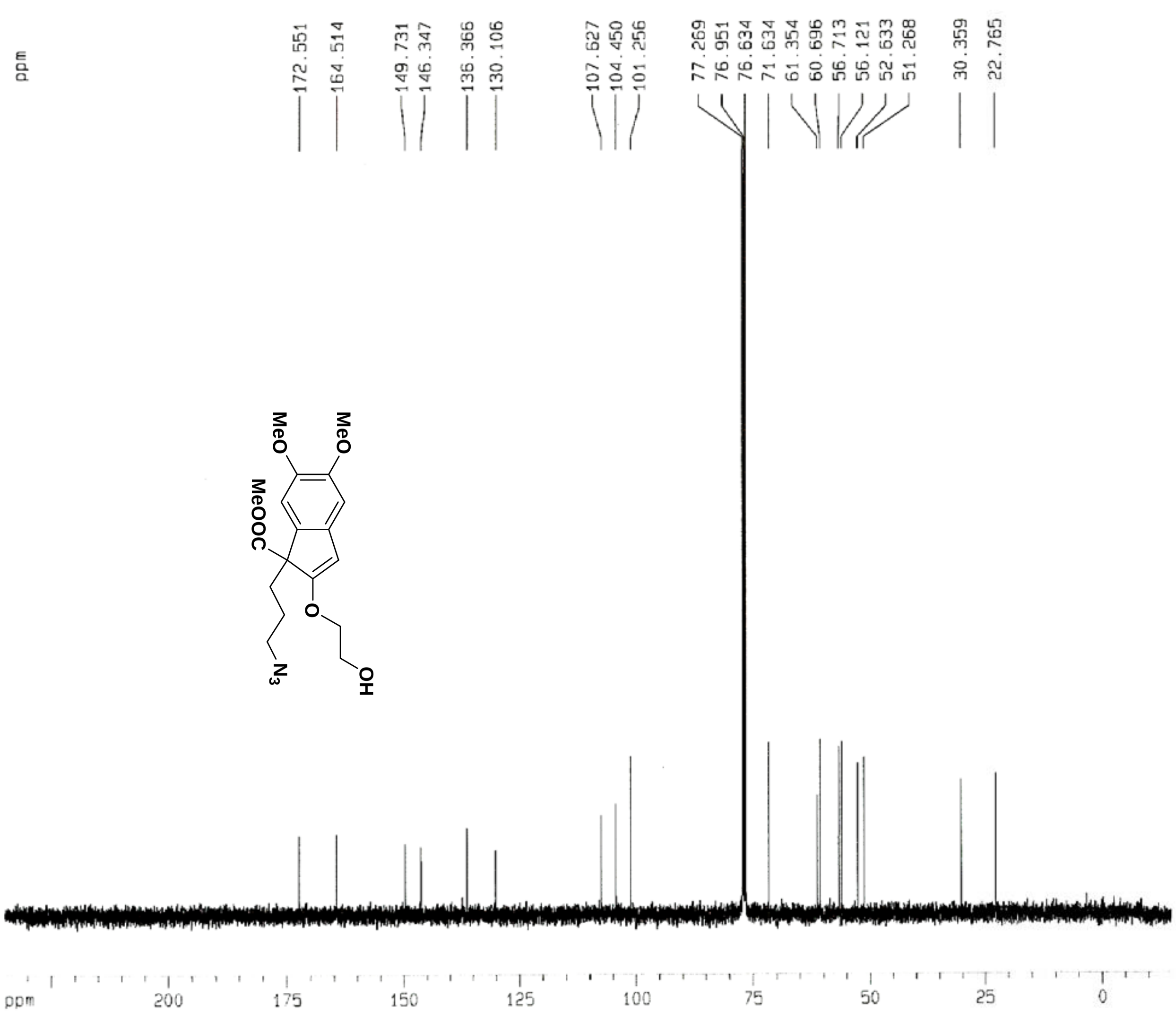


```
Current Data Parameters
NAME                sb2
EXPNO               622
PROCNO                1

F2 - Acquisition Parameters
Date_          20070608
Time              23.11
INSTRUM           av400
PROBHD   5 mm BBO BB-1H
PULPROG         zgpg30
TD                65536
SOLVENT           CDCl3
NS                 1000
DS                    2
SWH          25125.629 Hz
FIDRES        0.383387 Hz
AQ           1.3042164 sec
RG               1149.4
DW               19.900 usec
DE                 6.00 usec
TE                300.0 K
D1          2.00000000 sec
d11         0.03000000 sec
d12         0.00002000 sec

======== CHANNEL f1 ========
NUC1                13C
P1                11.00 usec
PL1                0.00 dB
SFO1        100.6238364 MHz

======== CHANNEL f2 ========
CPDPRG2         waltz16
NUC2                 1H
PCPD2             80.00 usec
PL2                3.00 dB
PL12              20.23 dB
PL13              16.00 dB
SFO2        400.1320007 MHz

F2 - Processing parameters
SI               131072
SF          100.6127690 MHz
WDW                  EM
SSB                   0
LB                 1.00 Hz
GB                    0
PC                 1.40

1D NMR plot parameters
CX                20.00 cm
CY                20.47 cm
F1P             234.849 ppm
F1             23628.84 Hz
F2P             -14.877 ppm
F2             -1496.79 Hz
PPMCM          12.48630 ppm/cm
HZCM         1256.28137 Hz/cm
```

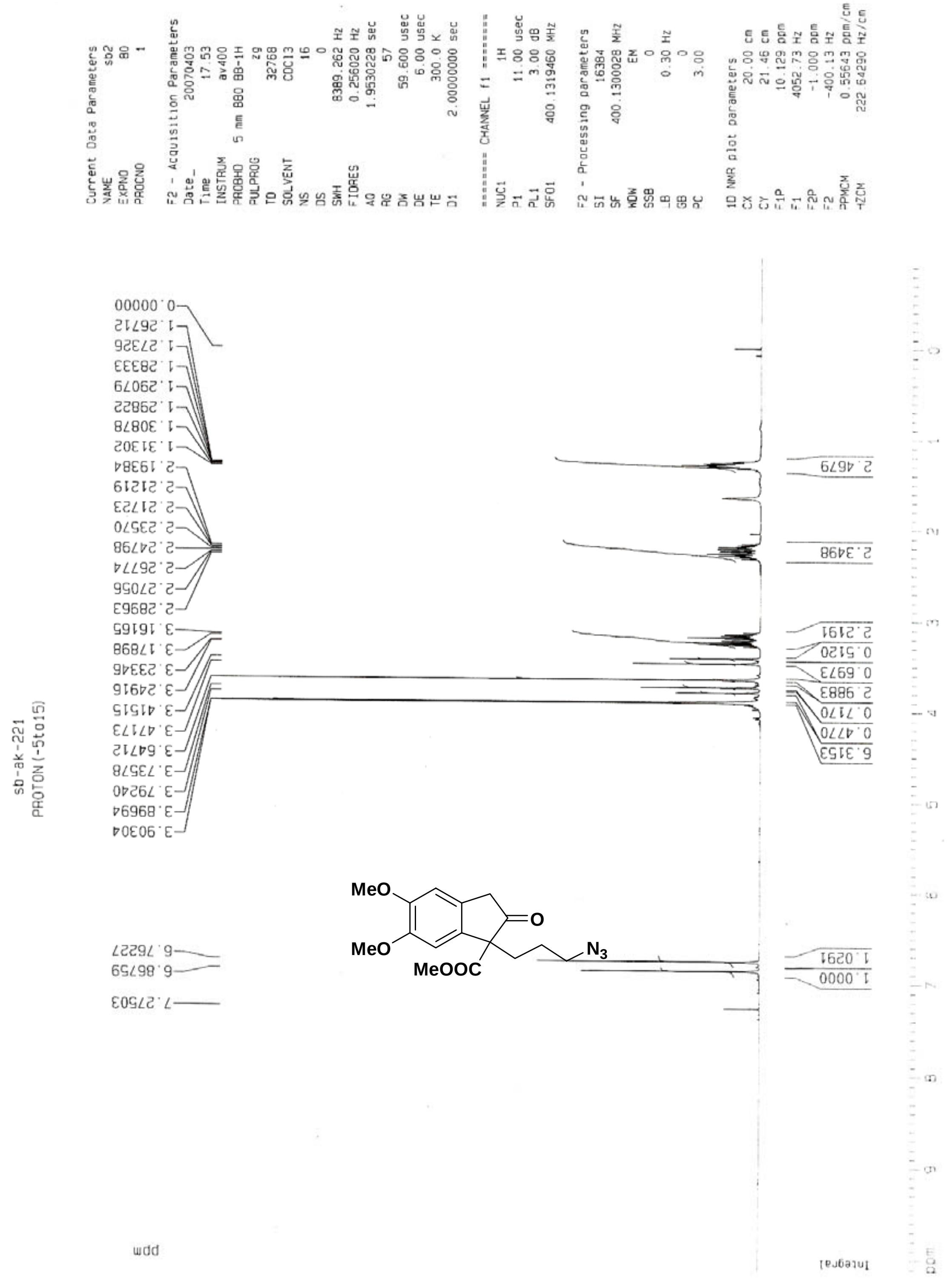
sb-ak-221
PROTON(-5to15)
0.00000
1.26712
1.27326
1.28333
1.29079
1.29822
1.30878
1.31302
2.19384
2.21219
2.21723
2.23570
2.24798
2.26774
2.27056
2.28963
3.16165
3.17898
3.23346
3.24916
3.41515
3.47173
3.64712
3.73578
3.79240
3.89694
3.90304
6.76227
6.86759
7.27503
ppm
MeO
MeO
O
MeOOC
N3
Current Data Parameters
NAME sb2
EXPNO 80
PROCNO 1
F2 - Acquisition Parameters
Date_ 20070403
Time 17.53
INSTRUM av400
PROBHD 5 mm BBO BB-1H
PULPROG zg
TD 32768
SOLVENT CDCl3
NS 16
DS 0
SWH 8389.262 Hz
FIDRES 0.256020 Hz
AQ 1.9530228 sec
RG 57
DW 59.600 usec
DE 6.00 usec
TE 300.0 K
D1 2.00000000 sec
======== CHANNEL f1 ========
NUC1 1H
P1 11.00 usec
PL1 3.00 dB
SFO1 400.1319460 MHz
F2 - Processing parameters
SI 16384
SF 400.1300028 MHz
WDW EM
SSB 0
LB 0.30 Hz
GB 0
PC 3.00
1D NMR plot parameters
CX 20.00 cm
CY 21.46 cm
F1P 10.129 ppm
F1 4052.73 Hz
F2P -1.000 ppm
F2 -400.13 Hz
PPMCM 0.55643 ppm/cm
HZCM 222.64290 Hz/cm
2.4679
2.3498
2.2191
0.5120
0.6973
2.9883
0.7170
0.4770
6.3153
1.0291
1.0000
Integral

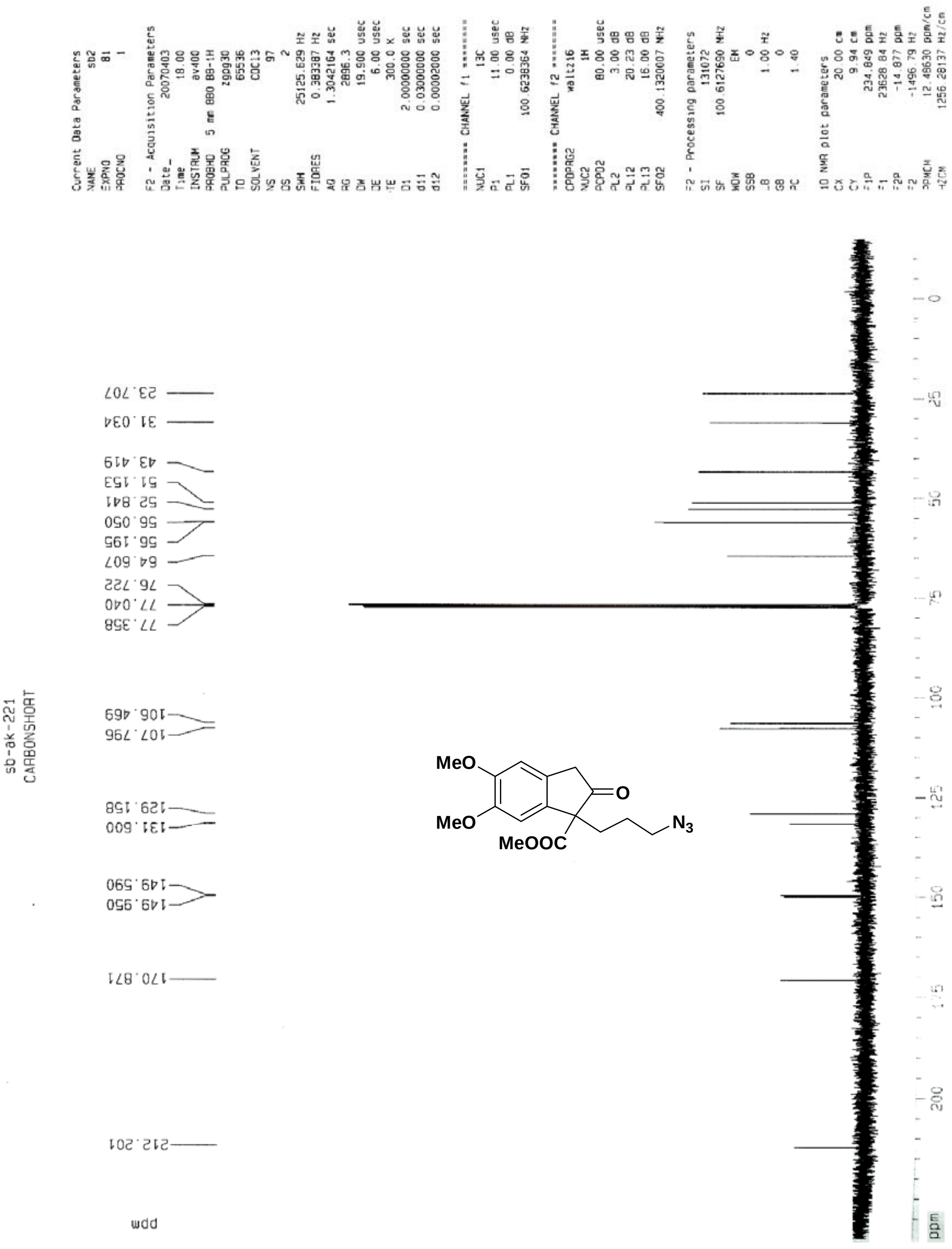
sb-ak-221
CARBONSHORT
23.707
31.034
43.419
51.153
52.841
56.050
56.195
64.607
76.722
77.040
77.358
106.469
107.796
129.158
131.600
149.590
149.950
170.871
212.201
ppm
MeO
MeO
O
MeOOC
N_3

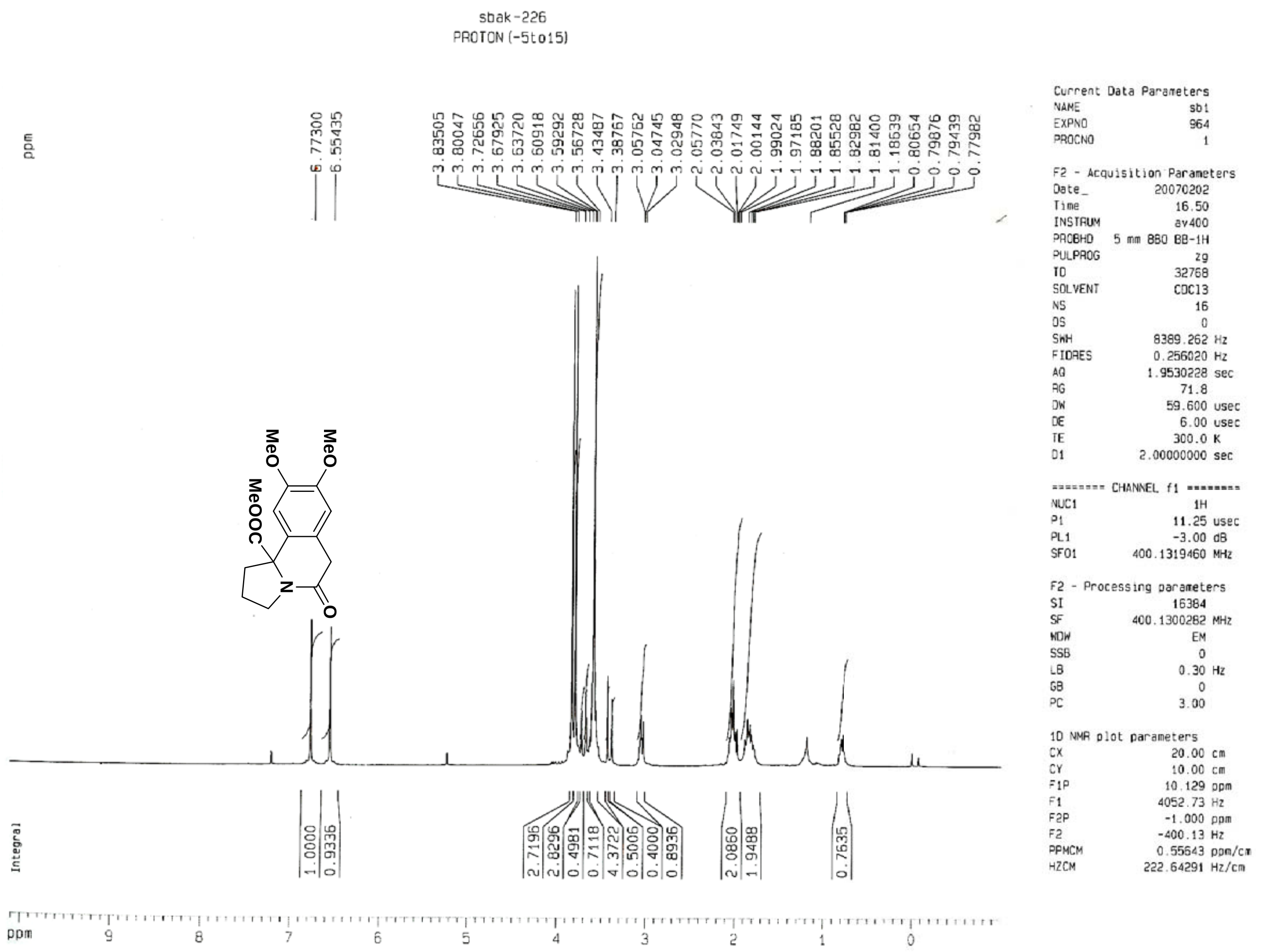
sbak-226
PROTON (-5to15)
MeO
MeO
MeOOC
N
O
6.77300
6.55435
3.83505
3.80047
3.72656
3.67925
3.63720
3.60918
3.59292
3.56728
3.43487
3.38767
3.05762
3.04745
3.02948
2.05770
2.03843
2.01749
2.00144
1.99024
1.97185
1.88201
1.85528
1.82982
1.81400
1.18639
0.80654
0.79876
0.79439
0.77982
Integral
1.0000
0.9336
2.7196
2.8296
0.4981
0.7118
4.3722
0.5006
0.4000
0.8936
2.0860
1.9488
0.7635
ppm
9
8
7
6
5
4
3
2
1
0
Current Data Parameters
NAME sb1
EXPNO 964
PROCNO 1
F2 - Acquisition Parameters
Date_ 20070202
Time 16.50
INSTRUM av400
PROBHD 5 mm BBO BB-1H
PULPROG zg
TD 32768
SOLVENT CDCl3
NS 16
DS 0
SWH 8389.262 Hz
FIDRES 0.256020 Hz
AQ 1.9530228 sec
RG 71.8
DW 59.600 usec
DE 6.00 usec
TE 300.0 K
D1 2.00000000 sec
======== CHANNEL f1 ========
NUC1 1H
P1 11.25 usec
PL1 -3.00 dB
SFO1 400.1319460 MHz
F2 - Processing parameters
SI 16384
SF 400.1300282 MHz
WDW EM
SSB 0
LB 0.30 Hz
GB 0
PC 3.00
1D NMR plot parameters
CX 20.00 cm
CY 10.00 cm
F1P 10.129 ppm
F1 4052.73 Hz
F2P -1.000 ppm
F2 -400.13 Hz
PPMCM 0.55643 ppm/cm
HZCM 222.64291 Hz/cm

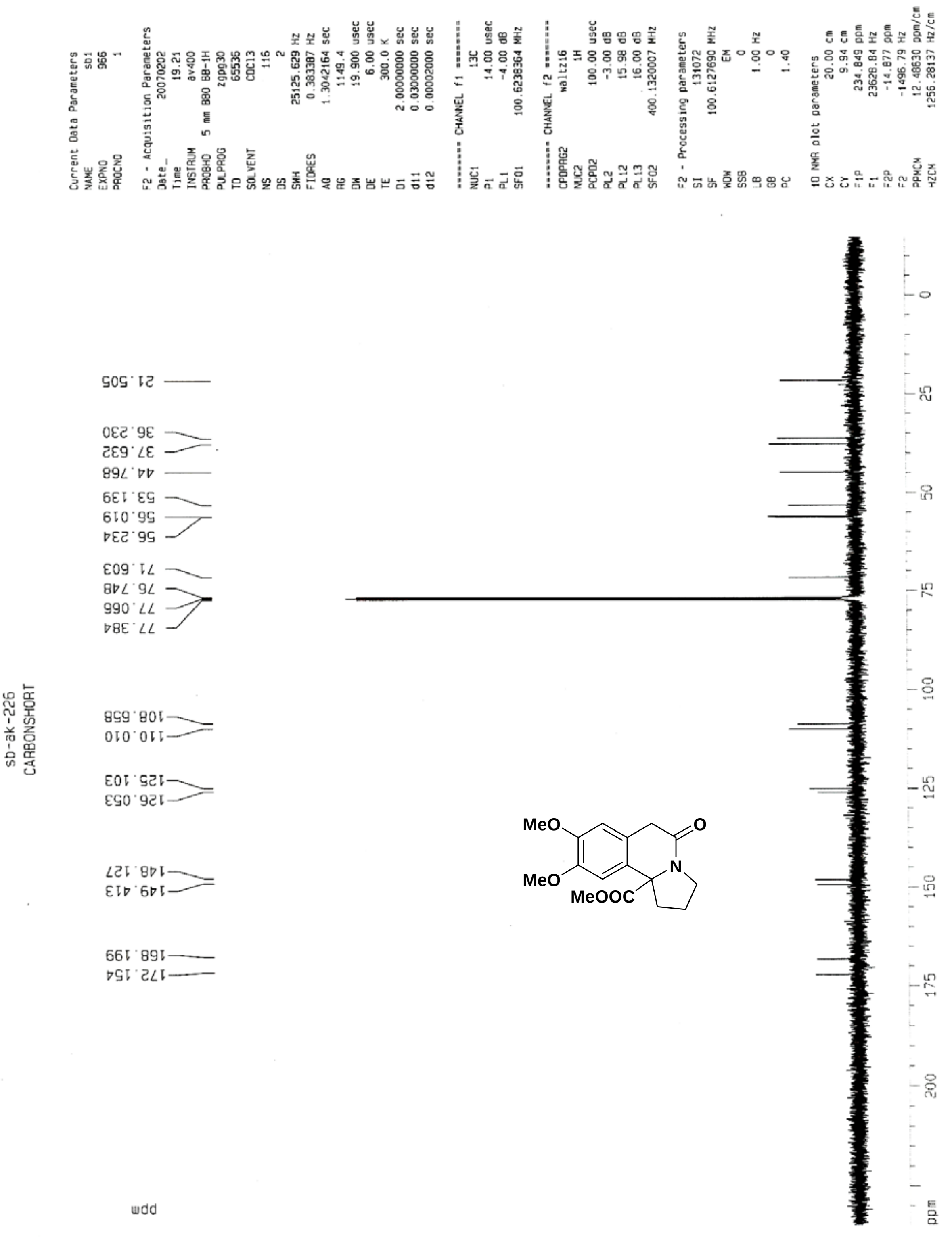
sb-ak-226
CARBONSHORT
21.505
36.230
37.632
44.768
53.139
56.019
56.234
71.603
76.748
77.066
77.384
108.658
110.010
125.103
126.053
148.127
149.413
168.199
172.154
ppm
MeO
MeO
MeOOC
O
N
Current Data Parameters
NAME sb1
EXPNO 966
PROCNO 1
F2 - Acquisition Parameters
Date_ 20070202
Time 19.21
INSTRUM av400
PROBHD 5 mm BBO BB-1H
PULPROG zgpg30
TD 65536
SOLVENT CDCl3
NS 116
DS 2
SWH 25125.629 Hz
FIDRES 0.383387 Hz
AQ 1.3042164 sec
RG 1149.4
DW 19.900 usec
DE 6.00 usec
TE 300.0 K
D1 2.00000000 sec
d11 0.03000000 sec
d12 0.00002000 sec
======== CHANNEL f1 ========
NUC1 13C
P1 14.00 usec
PL1 -4.00 dB
SFO1 100.6238364 MHz
======== CHANNEL f2 ========
CPDPRG2 waltz16
NUC2 1H
PCPD2 100.00 usec
PL2 -3.00 dB
PL12 15.98 dB
PL13 16.00 dB
SFO2 400.1320007 MHz
F2 - Processing parameters
SI 131072
SF 100.6127690 MHz
WDW EM
SSB 0
LB 1.00 Hz
GB 0
PC 1.40
1D NMR plot parameters
CX 20.00 cm
CY 9.94 cm
F1P 234.849 ppm
F1 23628.84 Hz
F2P -14.877 ppm
F2 -1496.79 Hz
PPMCM 12.48630 ppm/cm
HZCM 1256.28137 Hz/cm
0
25
50
75
100
125
150
175
200
ppm

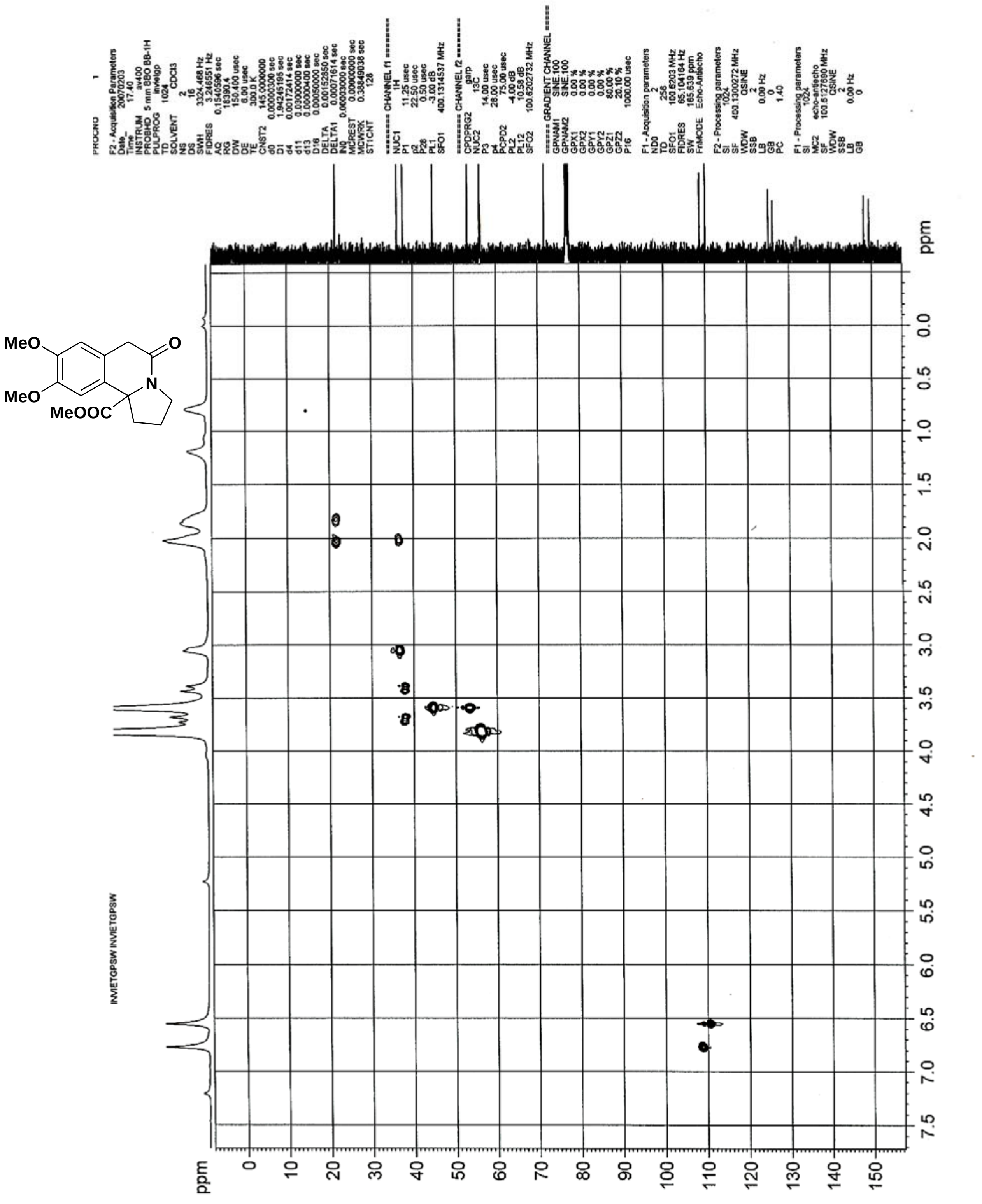
MeO
MeO
MeOOC
O
N
ppm

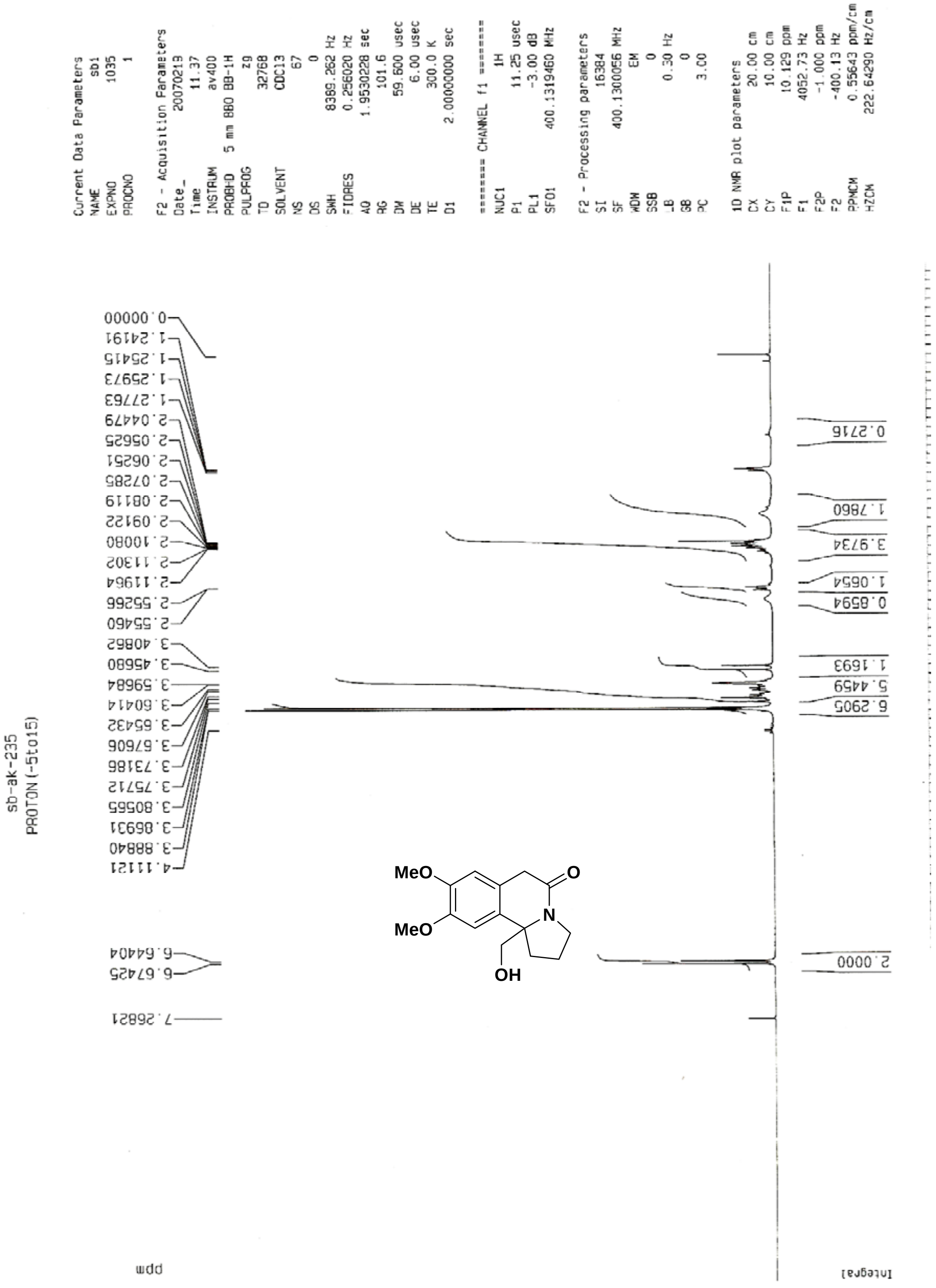
sb-ak-235
PROTON(-5to15)
Current Data Parameters
NAME sb1
EXPNO 1035
PROCNO 1
F2 - Acquisition Parameters
Date_ 20070219
Time 11.37
INSTRUM av400
PROBHD 5 mm BBO BB-1H
PULPROG zg
TD 32768
SOLVENT CDCl3
NS 67
DS 0
SWH 8389.262 Hz
FIDRES 0.256020 Hz
AQ 1.9530228 sec
RG 101.6
DW 59.600 usec
DE 6.00 usec
TE 300.0 K
D1 2.00000000 sec
======== CHANNEL f1 ========
NUC1 1H
P1 11.25 usec
PL1 -3.00 dB
SFO1 400.1319460 MHz
F2 - Processing parameters
SI 16384
SF 400.1300056 MHz
WDW EM
SSB 0
LB 0.30 Hz
GB 0
PC 3.00
1D NMR plot parameters
CX 20.00 cm
CY 10.00 cm
F1P 10.129 ppm
F1 4052.73 Hz
F2P -1.000 ppm
F2 -400.13 Hz
PPMCM 0.55643 ppm/cm
HZCM 222.64290 Hz/cm
0.00000
1.24191
1.25415
1.25973
1.27763
2.04479
2.05625
2.06251
2.07285
2.08119
2.09122
2.10080
2.11302
2.11964
2.55266
2.55460
3.40862
3.45680
3.59684
3.60414
3.65432
3.67606
3.73186
3.75712
3.80565
3.86931
3.88840
4.11121
6.64404
6.67425
7.26821
0.2716
1.7860
3.9734
1.0654
0.8594
1.1693
5.4459
6.2905
2.0000
Integral
ppm
MeO
MeO
O
N
OH

sb-ak-235
CARBONSHORT

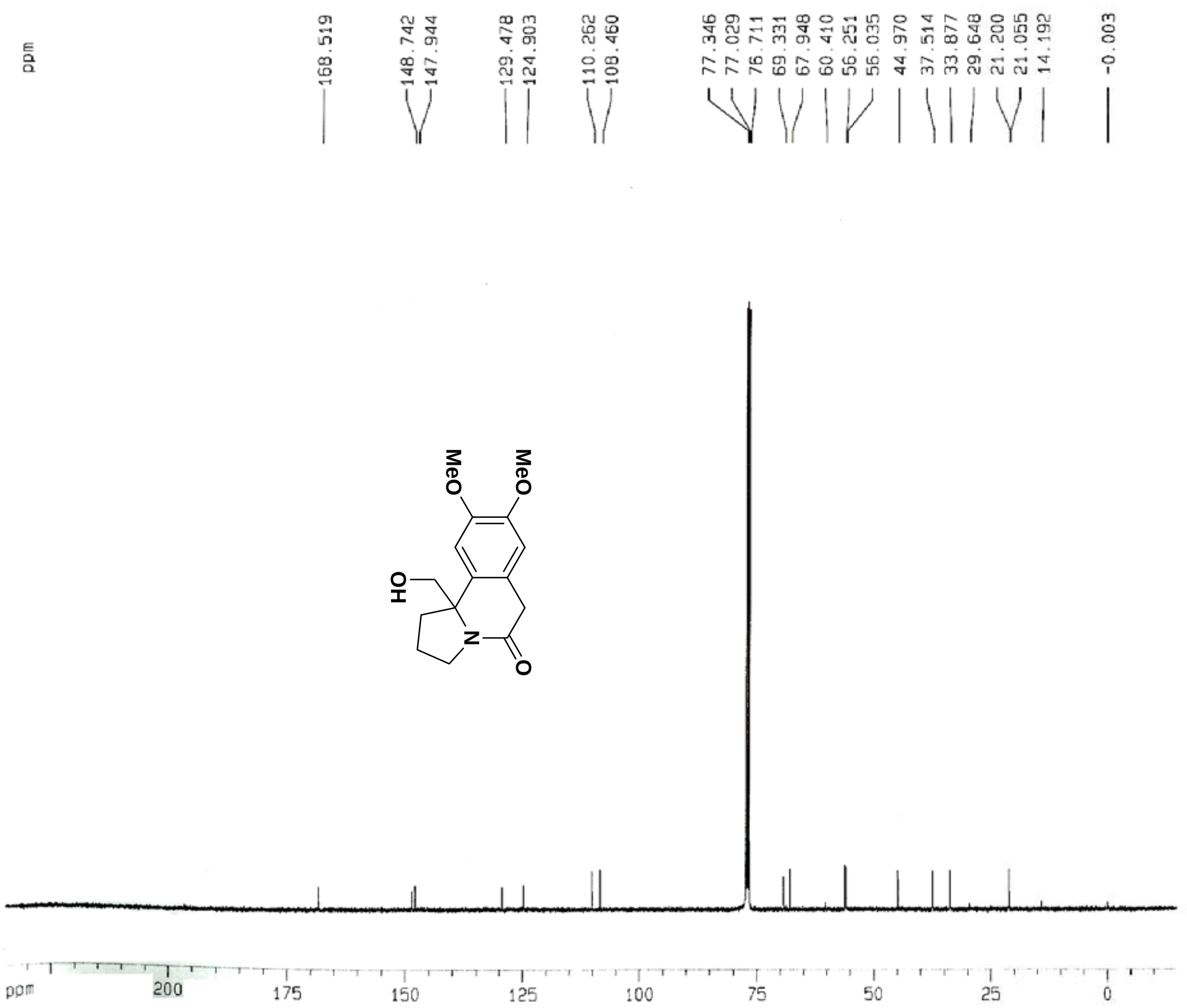

Current Data Parameters
NAME sb1
EXPNO 1031
PROCNO 1

F2 - Acquisition Parameters
Date_ 20070217
Time 9.04
INSTRUM av400
PROBHD 5 mm BBO BB-1H
PULPROG zgpg30
TD 65536
SOLVENT CDCl3
NS 8003
DS 2
SWH 25125.629 Hz
FIDRES 0.383387 Hz
AQ 1.3042164 sec
RG 1149.4
DW 19.900 usec
DE 6.00 usec
TE 300.0 K
D1 2.00000000 sec
d11 0.03000000 sec
d12 0.00002000 sec

======== CHANNEL f1 ========
NUC1 13C
P1 14.00 usec
PL1 -4.00 dB
SFO1 100.6238364 MHz

======== CHANNEL f2 ========
CPDPRG2 waltz16
NUC2 1H
PCPD2 100.00 usec
PL2 -3.00 dB
PL12 15.98 dB
PL13 16.00 dB
SFO2 400.1320007 MHz

F2 - Processing parameters
SI 131072
SF 100.6127690 MHz
WDW EM
SSB 0
LB 1.00 Hz
GB 0
PC 1.40

1D NMR plot parameters
CX 20.00 cm
CY 9.94 cm
F1P 234.849 ppm
F1 23628.84 Hz
F2P -14.877 ppm
F2 -1496.79 Hz
PPMCM 12.48630 ppm/cm
HZCM 1256.28137 Hz/cm

sb-ms-ak-mesylation
PROTON (-5to15)

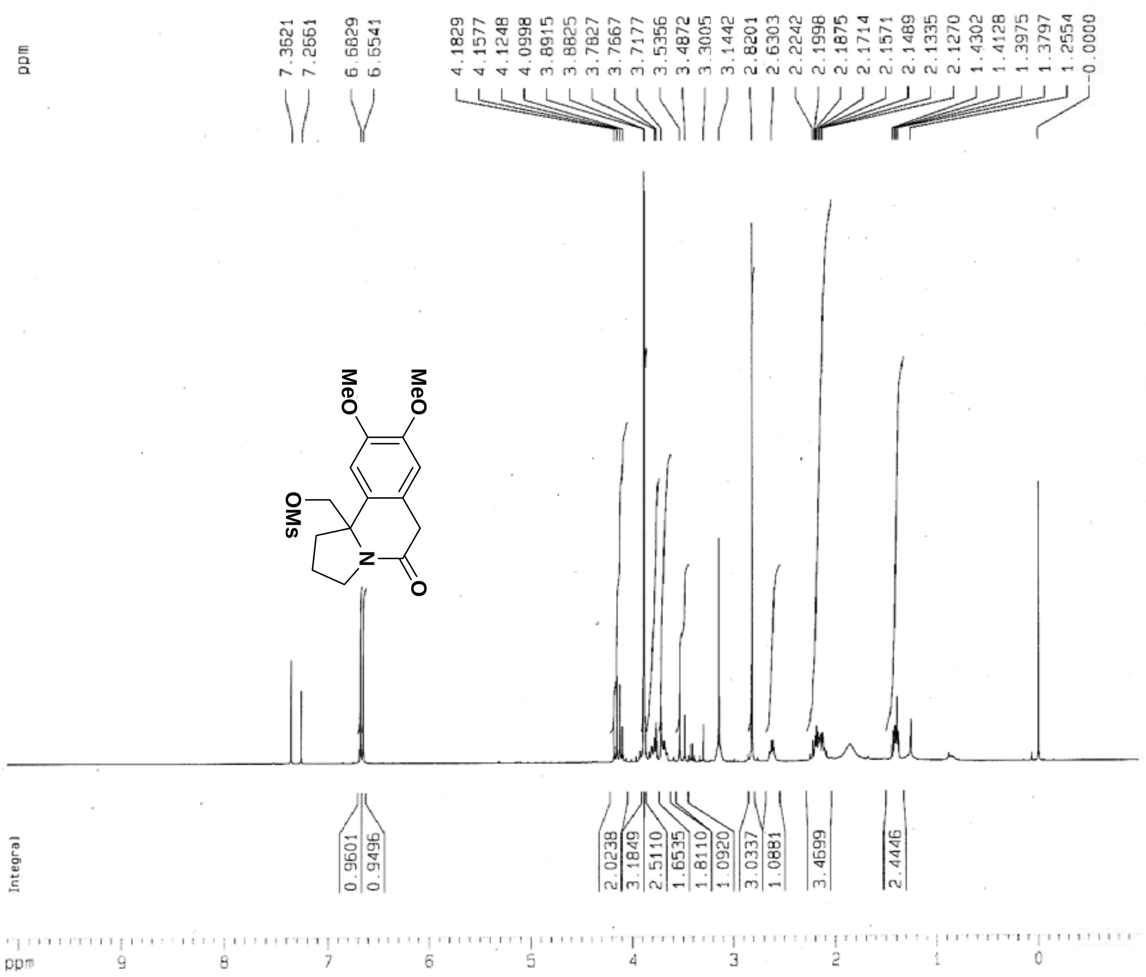

Current Data Parameters
NAME sb2
EXPNO 1316
PROCNO 1

F2 - Acquisition Parameters
Date_ 20070825
Time 9.04
INSTRUM av400
PROBHD 5 mm BBO BB-1H
PULPROG zg
TD 32768
SOLVENT CDCl3
NS 16
DS 0
SWH 8389.262 Hz
FIDRES 0.256020 Hz
AQ 1.9530228 sec
RG 128
DW 59.600 usec
DE 6.00 usec
TE 300.0 K
D1 2.00000000 sec

======== CHANNEL f1 ========
NUC1 1H
P1 11.00 usec
PL1 3.00 dB
SFO1 400.1319460 MHz

F2 - Processing parameters
SI 16384
SF 400.1300064 MHz
WDW EM
SSB 0
LB 0.30 Hz
GB 0
PC 3.00

1D NMR plot parameters
CX 20.00 cm
CY 10.00 cm
F1P 10.129 ppm
F1 4052.73 Hz
F2P -1.000 ppm
F2 -400.13 Hz
PPMCM 0.55643 ppm/cm
HZCM 222.64290 Hz/cm

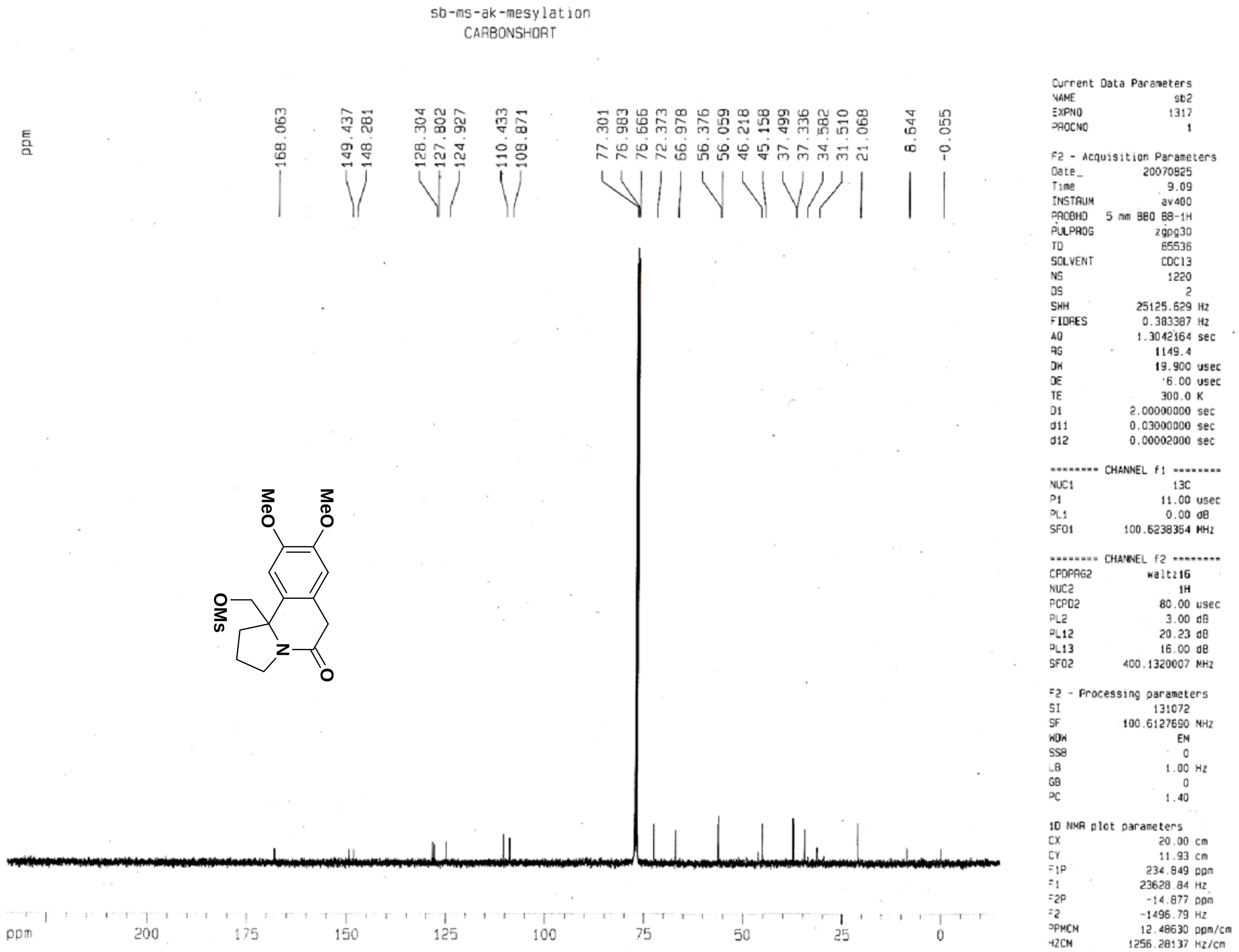
sb-ms-ak-mesylation
CARBONSHORT
168.063
149.437
148.281
128.304
127.802
124.927
110.433
108.871
77.301
76.983
76.666
72.373
66.978
56.376
56.059
46.218
45.158
37.499
37.336
34.582
31.510
21.068
8.644
-0.055
MeO
MeO
OMs
N
O
ppm
200
175
150
125
100
75
50
25
0
Current Data Parameters
NAME sb2
EXPNO 1317
PROCNO 1
F2 - Acquisition Parameters
Date_ 20070825
Time 9.09
INSTRUM av400
PROBHD 5 mm BBO BB-1H
PULPROG zgpg30
TD 65536
SOLVENT CDCl3
NS 1220
DS 2
SWH 25125.629 Hz
FIDRES 0.383387 Hz
AQ 1.3042164 sec
RG 1149.4
DW 19.900 usec
DE 6.00 usec
TE 300.0 K
D1 2.00000000 sec
d11 0.03000000 sec
d12 0.00002000 sec
======== CHANNEL f1 ========
NUC1 13C
P1 11.00 usec
PL1 0.00 dB
SFO1 100.6238364 MHz
======== CHANNEL f2 ========
CPDPRG2 waltz16
NUC2 1H
PCPD2 80.00 usec
PL2 3.00 dB
PL12 20.23 dB
PL13 16.00 dB
SFO2 400.1320007 MHz
F2 - Processing parameters
SI 131072
SF 100.6127690 MHz
WDW EM
SSB 0
LB 1.00 Hz
GB 0
PC 1.40
1D NMR plot parameters
CX 20.00 cm
CY 11.93 cm
F1P 234.849 ppm
F1 23628.84 Hz
F2P -14.877 ppm
F2 -1496.79 Hz
PPMCM 12.48630 ppm/cm
HZCM 1256.28137 Hz/cm
